# Spo0A∼P Imposes a Temporal Gate for the Bimodal Expression of Competence in *Bacillus subtilis*

**DOI:** 10.1371/journal.pgen.1002586

**Published:** 2012-03-08

**Authors:** Nicolas Mirouze, Yaanik Desai, Arjun Raj, David Dubnau

**Affiliations:** 1 Public Health Research Institute Center of New Jersey Medical School, Newark, New Jersey, United States of America; 2 School of Engineering and Applied Science, University of Pennsylvania, Philadelphia, Pennsylvania, United States of America; 3 Department of Microbiology and Molecular Genetics, University of Medicine and Dentistry of New Jersey, Newark, New Jersey, United States of America; Uppsala University, Sweden

## Abstract

ComK transcriptionally controls competence for the uptake of transforming DNA in *Bacillus subtilis*. Only 10%–20% of the cells in a clonal population are randomly selected for competence. Because ComK activates its own promoter, cells exceeding a threshold amount of ComK trigger a positive feedback loop, transitioning to the competence ON state. The transition rate increases to a maximum during the approach to stationary phase and then decreases, with most cells remaining OFF. The average basal rate of *comK* transcription increases transiently, defining a window of opportunity for transitions and accounting for the heterogeneity of competent populations. We show that as the concentration of the response regulator Spo0A∼P increases during the entry to stationary phase it first induces *comK* promoter activity and then represses it by direct binding. Spo0A∼P activates by antagonizing the repressor, Rok. This amplifies an *inherent* increase in basal level *comK* promoter activity that takes place during the approach to stationary phase and is a general feature of core promoters, serving to couple the probability of competence transitions to growth rate. Competence transitions are thus regulated by growth rate and temporally controlled by the complex mechanisms that govern the formation of Spo0A∼P. On the level of individual cells, the fate-determining noise for competence is intrinsic to the *comK* promoter. This overall mechanism has been stochastically simulated and shown to be plausible. Thus, a deterministic mechanism modulates an inherently stochastic process.

## Author Summary

Populations of bacterial cells sometimes bifurcate into subpopulations with different patterns of gene expression. The soil bacterium *B. subtilis* becomes “competent” for the uptake of environmental DNA, thus acquiring new genetic information. About 15% of the cells are chosen for expression of the competence genes by stochastic fluctuations in the transcription of *comK*. When the concentration of ComK exceeds a critical threshold, it activates its own expression, a molecular switch is thrown, and competence ensues in that cell. Here we ask why all of the cells do not eventually throw the switch. We show that the basal level expression of *comK* increases and then decreases as nutrients are exhausted, so that the number of cells exceeding the ComK threshold rises and falls, opening and closing a window of opportunity for competence. Two factors responsible for this “uptick” in *comK* expression are: 1) a global increase in transcription as cell division slows, and 2) a continual rise in the concentration of the master regulatory protein Spo0A-P, which activates and then represses *comK* as it accumulates. The global increase transmits growth rate information and the increase in Spo0A∼P encodes multiple signals, including the nutritional, replication, and population density status of the culture.

## Introduction

Stochastic gene expression during development has received much attention in both bacterial and eukaryotic systems [1], [2], [3]. The developmental choices exhibited by the model bacterium *Bacillus subtilis* have emerged as favorite subjects for the analysis of stochastic decisions [4], [5], [6], [7], [8], [9], [10], [11]. When faced with the exhaustion of nutrients, individual bacteria may sporulate, become “competent”, or may form multi-cellular communities (biofilms) [12], [13], [14], [15]. Remarkably, all of these adaptive responses are expressed heterogeneously in populations. For example, only about 10–20% of the cells in a culture express the proteins needed for the uptake of DNA [16], [17]; most of the cells therefore remain in the non-competent state. The selection of cells for competence is random, driven by noise in the expression of a transcription factor gene, *comK* [5], [6], [8].

ComK is the proximal regulator of competence gene expression. This protein not only activates transcription of the downstream genes that encode DNA-uptake proteins, but also positively regulates its own promoter, and activation of the resulting positive feedback loop is at the heart of the decision to become competent [18], [19], [20]. Cells with more than a threshold amount of ComK induce further expression of *comK* and as a result can express the downstream genes. Explicit models have been suggested to explain the behavior of this system and experimental evidence has served to test and confirm the general validity of the models, particularly the central roles of positive feedback, a thresholded response to ComK and of noise in *comK* expression [5], [6], [8], [19], [20], [21]. During long-term cultivation, cells have been observed to enter and then exit the competent state, prompting the description of competence as an excitable system [5]. In bulk cultures grown to saturation, the *rate* of transitions to the competence-ON state increases rapidly as the culture approaches stationary phase and then decreases, approaching zero 1–2 hours later, so that most cells never express competence genes [8], [11], [22]. An explanation for this “window of opportunity” was provided by the observation that the basal level of *comK* mRNA increases during growth and then decreases, reaching a maximum level of 1–2 transcripts per cell near the time of the highest transition rate [8], [11]. Thus as the average mRNA content (and therefore the average ComK content) of the population increases, more cells cross the threshold and become competent. As the rate of transcription then approaches zero and *comK* transcripts decay, the average ComK content per cell no longer increases, or does so slowly. Cells that were already across the threshold would become competent with high probability and cells below the threshold would tend to remain in the OFF state. The increase and decrease in *comK* promoter activity (referred to hereafter as the “uptick”) was thus assigned the important role of defining the window of opportunity for making competent cells. In other words, a programmed regulatory change would adjust the probability of this developmental transition, modulating a stochastic decision-making process.

We show here that the phosphorylated form of the response-regulator protein Spo0A both activates and represses the basal firing rate of P*comK* and does so by direct binding to a series of activator and operator sites. Hence, the gradual increase in Spo0A∼P (OA∼P throughout) as cells enter stationary phase, which is documented in this report, influences the uptick profile of P*comK* activity. Remarkably, 0A∼P acts on P*comK* by amplifying a general increase in the rate of transcription also characteristic of synthetic “core” promoters containing only the −35 and −10 RNA polymerase (RNAPol) recognition motifs, which takes place as growth slows. This amplification is largely due to the action of 0A∼P as an anti-repressor toward the repressor protein Rok. As predicted by this mechanism, we show that a cell destined for competence must have a level of 0A∼P within a certain permissive range; below and above this range the probability of transition is low. We also show that noise in the level of 0A∼P within the permissive range makes a relatively small contribution to the determination of which cells transition to competence. This choice depends instead on intrinsic cell-to-cell variation in the firing of P*comK*. We propose that an extrinsically determined increase in the average firing rate of core promoters and a programmed increase in 0A∼P concentration define the window of opportunity for entrance to the competent state.

## Results

### The uptick of P*comK* activity is controlled by Rok and 0A∼P

**Figure 1:**
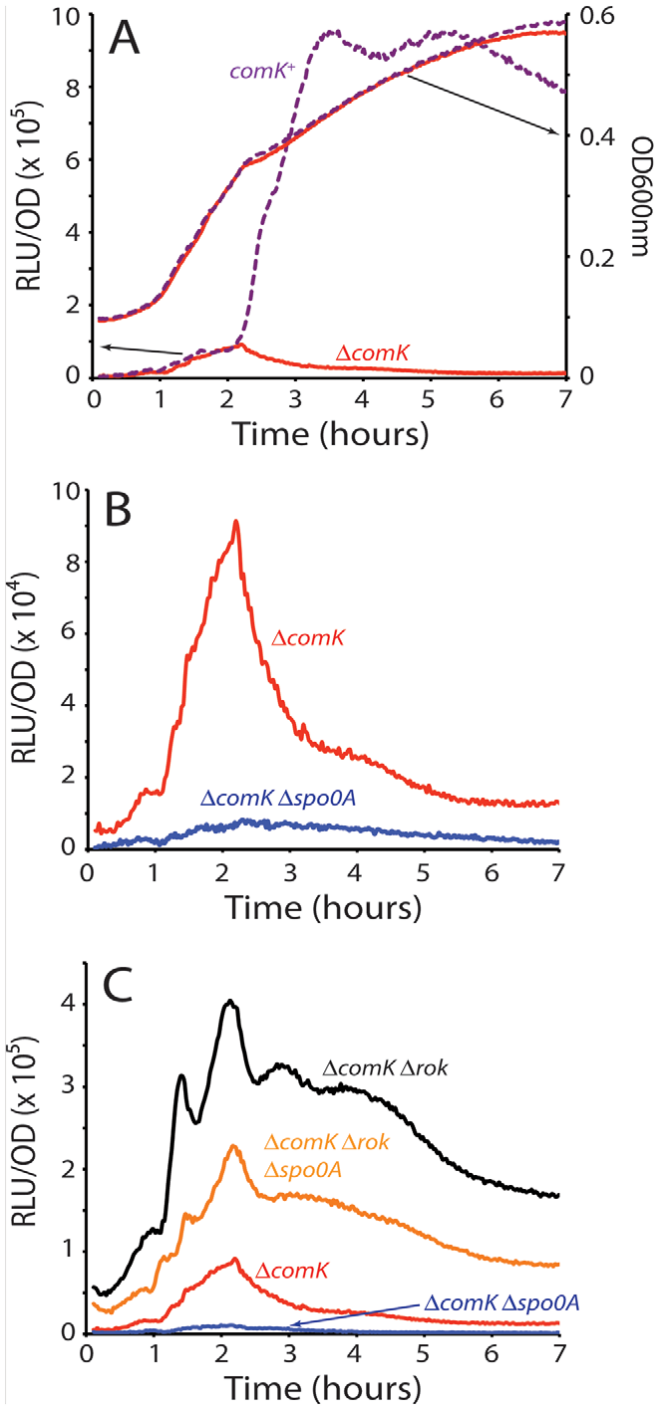
Transcription rates from the *comK* promoter. (A) The relative luminescence readings corrected for OD for the *comK* promoter and the OD readings for the growth curves are presented for the wild type (purple dotted curves) and *ΔcomK* (red curves) strains. Each pair of curves is connected to its Y-axis by a black arrow. (B) Expression from the comK promoter in a *ΔcomK* background compared with expression from the same promoter in a *ΔcomK Δspo0A* background. (C) Expression from P*comK* in *ΔcomK*, *ΔcomK Δspo0A*, *ΔcomK Δrok* and the *ΔcomK Δrok Δspo0A* backgrounds.

Based on measurements in single cells, we previously reported an uptick in the content of *comK* transcripts and it has been proposed that this variation determines the probability of transition to the competent state [8], [11]. Figure 1A shows that the uptick is also evident from ensemble measurements, using a fusion of P*comK* to the firefly luciferase coding sequence (*luc*). For this, the cultures were grown in a temperature-controlled plate reader in the presence of luciferin, as previously described [23], a method characterized by remarkable reproducibility (Figure S1). It is important to recognize that luciferase activity reports the transcription rate, rather than the cumulative activity of P*comK* [23]. As shown in Figure 1A and 1B, and confirming the previous result obtained with fluorescence *in situ* hybridization, the uptick is independent of *comK*. Thus, the basal level of *comK* promoter activity increases in the null-mutant *comK* strain during growth, reaching a maximum when the growth rate decreases, and thereafter declines. In a *comK* ^+^ strain, the initial rise is identical, but as the positive feedback is activated and cells transition to competence expression, a massive induction of P*comK* takes place, obscuring the decline in the basal level expression that is known to take place in the non-competent cells [8], [11].

**Figure 2:**
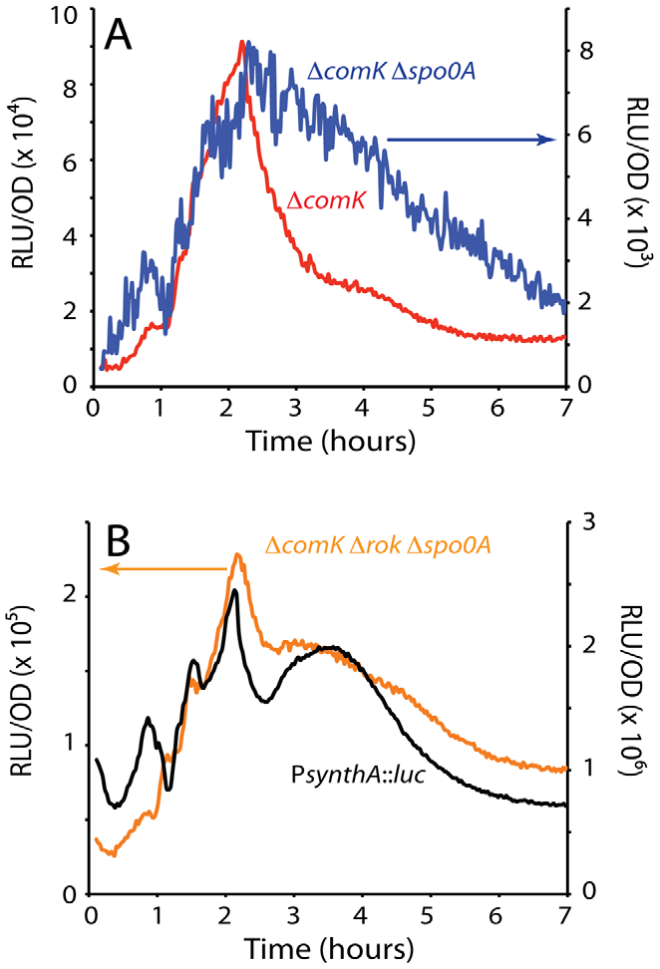
*comK* expression in the absence of Spo0A and expression of a synthetic promoter (P*synthA*) containing the −35 and −10 motifs of SigA-dependent promoters. (A) Expression from P*comK* in a *ΔcomK* background compared with the expression in a *ΔcomK Δspo0A* strain (see Figure 1B). The scale on the left of the graph is used to plot the data from the *ΔcomK Δspo0A* strain. (B) The expression from P*comK* in a *ΔcomK Δrok Δspo0A* background is plotted in parallel with the expression from a ‘core’ P*synthA* promoter. The scale on the left applies to P*comK*. The P*synthA-luc* fusion was placed at the ectopic *amyE* locus.

Using the P*comK-luc* fusion in a *comK* null-mutant strain, we adopted a candidate approach to determine factors involved in the uptick, introducing null-mutations in each of the genes known to encode regulatory factors for competence; *rok*, *spo0A*, *degU*, *abrB*, *codY* and *sinR*. Although several of these mutations slightly modified the amplitude of the uptick (not shown), only *spo0A* and *rok* exhibited major effects. In a *spo0A* null background (Figure 1B and 1C), the amplitude of the uptick was decreased about 9-fold, consistent with the observation that a *spo0A* loss-of-function mutant does not express competence genes [24]. The negative effect of the *spo0A* null mutant was not reversed by inactivation of abrB, showing that it is not due to over-expression of this repressor (not shown). When plotted to equalize the peaks of the s*po0A* ^+^ and Δ*spo0A* strains, the increasing portions of the uptick in the two strains are closely similar (Figure 2A). In both strains the downward portion begins at the same time, but that of the s*po0A* ^+^ strain is more abrupt (Figure 2A). It appears that Spo0A amplifies an underlying increase in the transcription rate of *comK* and also contributes to the relatively sharp decrease in the transcription rate that normally takes place. Thus, we predict that OA∼P may exert both positive and negative effects on P*comK*, depending on its time of action. All of the known activities of Spo0A are dependent on its phosphorylation by a phosphorelay, in which several kinases feed phosphoryl groups to a cascade of three proteins, the last of which is Spo0A [25]. The activity of Spo0A just described is no exception; inactivation of a phosphorelay gene (*spo0F*) and of two kinases (*kinA* and *kinB*) decreases the uptick amplitude (Figure S2).

As previously reported, in the absence of Rok, a repressor of P*comK* [26], the amplitude of the uptick was enhanced markedly but the timing of its onset was not altered [8], [26]. (Compare the Δ*comK* and Δ*comK* Δ*rok* strains in Figure 1C.) The increased amplitude is consistent with the published observation that the fraction of competent cells increases to 70–80% in a Δ*rok comK* ^+^ strain, while the time of transition to competence is not changed [26]. Because the average basal level of *comK* expression is increased in the *rok* strain, more cells exceed the threshold for competence. Importantly, when *rok* was inactivated in the Δ*comK* Δ*spo0A* background, a dramatic recovery of P*comK-luc* activity was observed, to about 60% of the level of the Δ*comK* Δ*rok spo0A^+^* strain (Figure 1C). This recovery suggests that 0A∼P may act at least in part by antagonizing the action of Rok. These changes in Pc*omK* activity were not due to an effect of *spo0A* inactivation on the amount of Rok protein (Figure S3) and we show below that they are explained instead by direct interactions of Rok and 0A∼P with the promoter of comK. In the two Δrok strains shown in Figure 1C the decreasing segment of the expression profiles was altered by the appearance of a pronounced shoulder. The evidence presented so far suggests that Rok and 0A∼P contribute to the normally abrupt downturn in transcription rate from P*comK* as well as acting respectively as repressor and activator of P*comK* earlier in the development of competence.

We wondered if the changes in P*comK* expression observed even in the Δ*spo0A* strain (Figure 2A) were specific to the *comK* promoter or were more global in nature. To answer this question we tested a synthetic SigA-dependent promoter derived from random sequences, except for the presence of canonical −35 and −10 motifs (P*synthA*) (Figure S4). This promoter was fused to the *luc* coding sequence, preceded by the ribosomal binding site from the *spoVG* gene. Figure 2B shows that P*synthA* exhibited an expression profile very similar to that of *PcomK* in the Δ*comK* Δ*rok* Δ*spo0A* background. A similar result was obtained using a previously described [23] fusion of *luc* to a stripped-down promoter consisting of the −35 and −10 sequences of the *spo0A* vegetative (SigA-dependent) promoter (not shown). We have also constructed a synthetic promoter carrying Sigma H motifs (P*synthH*) and fused this to *luc*. This promoter also showed a pattern like that of the P*synthA* promoter (Figure S4). We conclude that P*comK* exhibits a change in activity during the transition to stationary phase, which is a general characteristic of promoter core elements. It also appears that Spo0A and Rok modulate this activity change in the case of P*comK*, causing changes in amplitude and collaborating to suppress the shoulder that occurs in the core promoters during the downward response. We suspect that these core promoter changes reflect global metabolic changes during the slowing of growth as cells approach stationary phase, perhaps due to the release of RNAPol from the transcription of stable RNA genes [23].

### Structure of the *comK* promoter region

**Figure 3:**
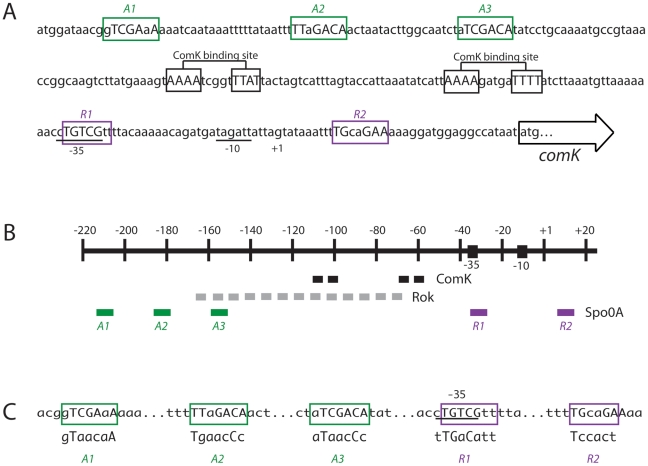
Organization of the *comK* promoter region. (A) Putative Spo0A binding sites for activation (*A1*, *2* and *3*) or inhibition (*R1* and *2*) are shown, as are the two ComK binding sites [27], the −35 and −10 promoter motifs, the start site (+1) of transcription and the initiation codon for translation. The Spo0A binding sites are predicted based on the published consensus [30]. (B) Schematic representation of the *comK* promoter showing the putative Spo0A binding sites in relation to the ComK boxes and the region in which Rok binds [28]. (C) Mutagenesis of the Spo0A binding sites. For each box the mutagenized sequences are shown below the wild type sequences. In panels A and C residues that differ from the Spo0A binding consensus sequence are shown in lower case.

These effects of *rok* and *spo0A* on P*comK* activity prompted examination of the sequence surrounding P*comK* (Figure 3). ComK is known to act positively on its own promoter, interacting with ComK boxes located upstream of the −35 SigA recognition motif [27], while Rok represses P*comK* by direct binding [26]. Rok is known to interact at least within an approximately 100 bp region (Figure 3B) [28], which overlaps the ComK boxes and has been shown to bind preferentially to AT-rich sequences [29]. ComK activates P*comK* largely as an anti-repressor for Rok, although it does not displace this repressor from the DNA [28].

Inspection of the region surrounding P*comK* revealed five sequences resembling the Spo0A binding consensus [30], which are illustrated in Figure 3A and 3B. The positions of these sequences suggested that three of them (A1, A2 and A3) functioned as activation sites and that the remaining two (R1 and R2) were sites for repression. None of these putative binding motifs have more than 2 out of 7 differences from the consensus and *A1* and *3* have one mismatch each. The presence of the putative 0A∼P binding sites and their locations suggested a role for increasing concentrations of 0A∼P, activating at *A1*, *2* and *3* and then repressing at *R1* and *2*. Such a mechanism would resemble the action of 0A∼P at the promoter of *sinI* [31] although in the present case we must also posit a regulatory role for Rok. This model requires that 0A∼P binds directly to P*comK* at the sites we have identified. We were encouraged by a previous observation that Spo0A was able to bind to a *comK* promoter fragment [30]. Accordingly, we next determined whether Spo0A does indeed bind to *A1, 2, 3* on one hand, and to *R1* and *R2* on the other.

### 0A∼P binds directly to P*comK*

**Figure 4:**
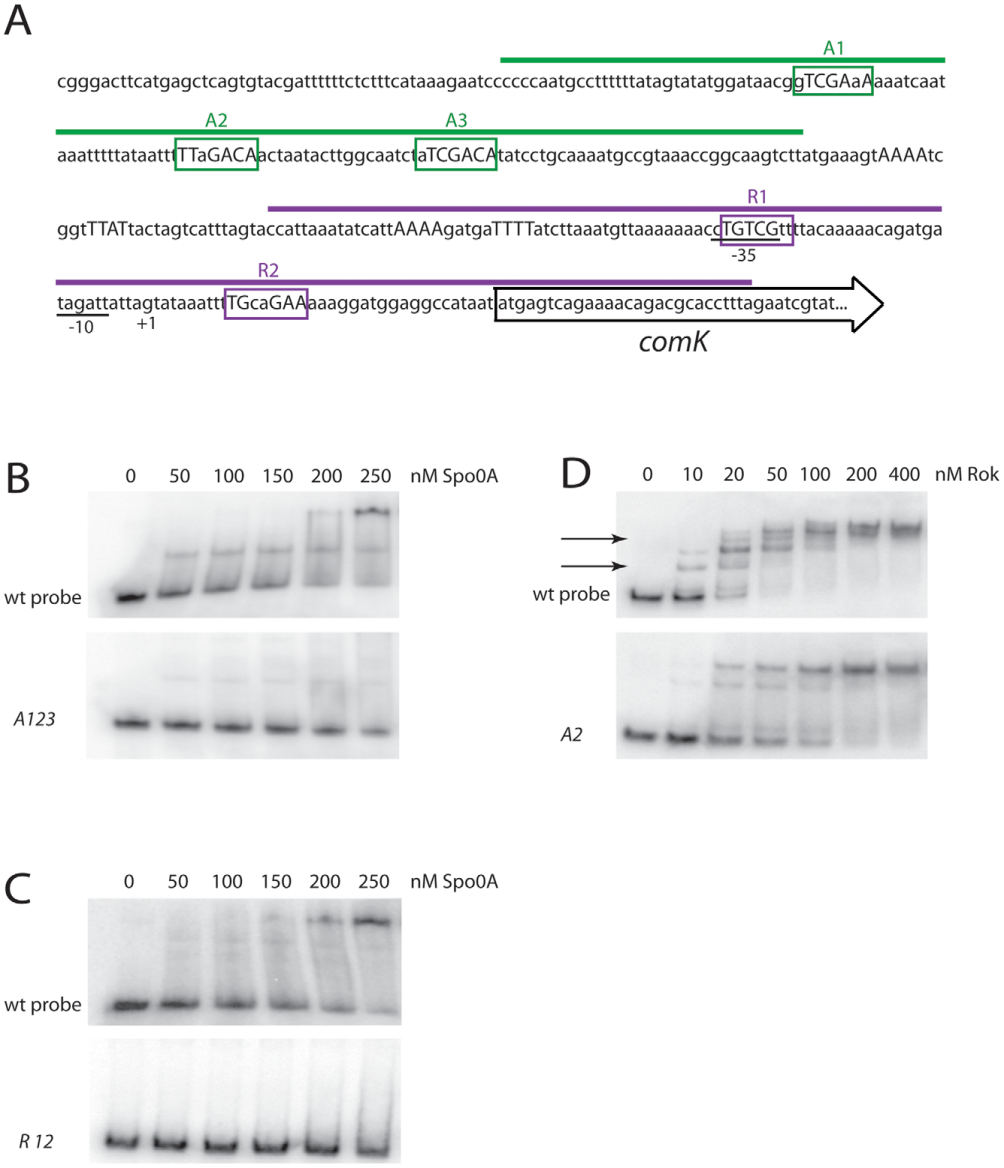
Spo0A binds directly to sequences in the *comK* regulatory region. (A) The P*comK* sequence is displayed with the extents of the wild type probes encompassing *A1*, *2* and *3* (green) and *R1* and *2* (purple) indicated. (B) A radiolabeled DNA fragment containing *A1*, *2* and *3* was incubated with the indicated concentrations of 0A∼P and the mixture was resolved by polyacrylamide gel electrophoresis. In the lower section of the panel, an otherwise identical probe with the *A2*, *2* and *3* mutations (Figure 3) was used. (C) In this panel the probe fragment in the upper gel contained *R1* and *R2*. The probe in the lower section carried *R1* and *R2* mutations (Figure 3). (D) The wild type probe used in panel B was incubated with Rok at the indicated concentrations and the results of gel electrophoresis are shown in the upper panel. In the lower panel an identical probe was used in which the A2 mutation diagrammed in Figure 3C was introduced.

**Figure 5:**
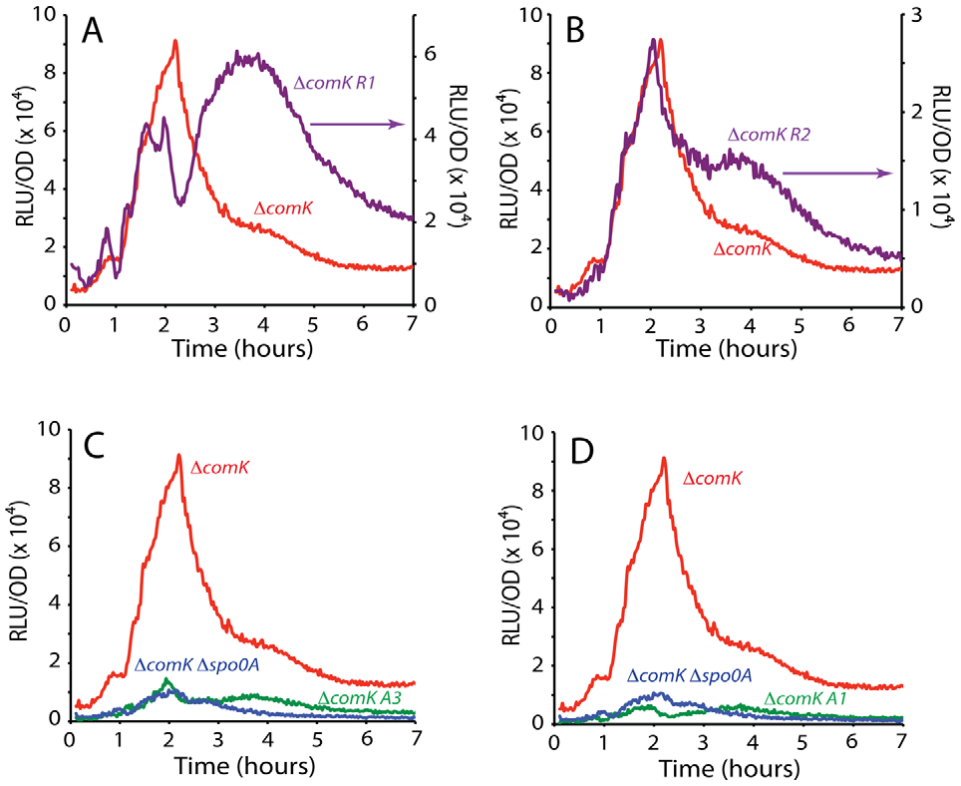
Effect of mutations in the putative Spo0A binding sites on *comK* expression. The effects of mutations in *R1* (panel A), *R2* (panel B), *A3* (panel C) or *A1* (panel D) in the *ΔcomK* background are compared with the expression from the wild type *comK* promoter.

**Figure 6:**
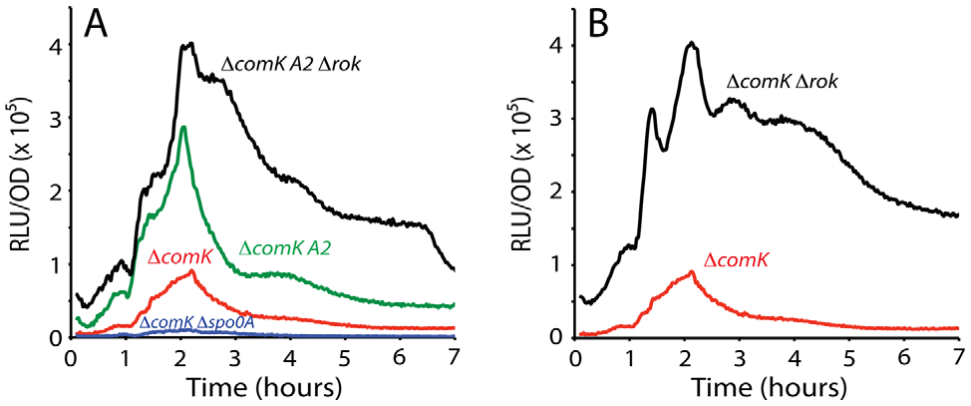
Rok and Spo0A bind at *A2*. Panel A shows the effect of an *A2* mutation on *comK* expression, alone and in combination with a null-mutation in *rok*. The effect of a *spo0A* knockout mutation is shown for comparison and panel B compares the effect of a *rok* mutation alone.

To test the binding of Spo0A to sequences surrounding P*comK*, we utilized gel shifts of radiolabeled DNA fragments incubated with purified full length 0A∼P. Figure 4B shows the results of an experiment using a 128 bp fragment (Figure 4A) containing *A1*, *2* and *3* and an identical experiment with the same fragment in which the putative Spo0A binding sites carried the triple *A123* mutations described above (Figure 3C). With the wild type fragment, 50% of the probe was shifted by a Spo0A concentration of about 50 nM. Due to uncertainty concerning the fraction of phosphorylated versus unphosphorylated protein, this is likely to be an overestimate of the nominal K_D_. The mutant fragment exhibited little or no shift over the protein concentration range used for this experiment. These results strongly suggest that 0A∼P binds to P*comK* directly, and that the sequences identified as *A1*, *2* and *3* are important for binding, in agreement with the *in vivo* data in Figure 5 and Figure 6.

A similar experiment (Figure 4C) was carried out using a 146 bp fragment (Figure 4A) containing *R1* and *2* as well as an identical fragment with the mutations that were shown to inactivate these sites *in vivo* (Figure 3, Figure 5E and 5F). A shift was noted with the wild type fragment, with a nominal K_D_ of 100–150 nM. Once again the mutant fragment exhibited little or no evidence of binding.

To test whether A2 is a binding site for Rok, a gel shift experiment was carried out using the wild type 128 bp probe fragment also used in Figure 4B. Multiple shifted bands were detected (Figure 4D, upper panel) with a K_D_ in the low nanomolar range, consistent with results obtained with other promoters [26]. The A2 mutation indicated in Figure 3C was introduced into this probe and the gel shift results obtained with Rok (Figure 4D, lower panel) permit two conclusions. First, the nominal K_D_ is shifted from about 15 nM to about 50 nM, and two shifted bands, indicated by arrows in the figure are missing with the mutant probe consistent with a high affinity interaction of Rok with A2. Second, even with A2 inactivated, residual lower affinity interaction of Rok to this probe remains, suggesting that Rok interacts with additional nearby sites.

### Regulation at P*comK*

As a first test of the roles of the putative OA∼P binding sites, we introduced mutations (Figure 3C) and determined their effects on P*comK-luc* expression in a *comK* background. Because *R1* overlapped the −35 motif we changed this motif to the consensus TTGACA to avoid inactivating the promoter, leaving only three out of seven bases of the −35 consensus. The other mutant Spo0A boxes retained two out of seven consensus bases.

Inactivation of either *R1* or *R2* introduced prominent shoulders in the declining portion of the uptick profile, without changing the timing of the increasing segment (Figure 5A and 5B), supporting a role for these sequences in closing the window of opportunity. Although the amplitude of the *R1* shoulder is greater than that of *R2*, the timings of the increases and decreases in the two profiles are similar. These profiles also resemble those of the various Δ*rok* and Δ*spo0A* constructs and of the P*synthA-luc* and P*synthH* fusions (Figure 1C, Figure 2A and 2B, Figure S4). Figure S5 shows a comparison of the declining portions of the *R1*, *R2*, P*synthA* and wild type promoters and the effects of the Δ*rok*, Δ*spo0A* and combined Δ*rok* and Δ*spo0A* mutations, with their peak values normalized. The roughly similar overall shapes of these curves are consistent with the predicted role for 0A∼P as a repressor that interacts with *R1* and *2*, collaborating with Rok in suppressing a continuation of P*comK* activity. The observation that inactivation of either *spo0A* or *rok* gives a similar slow decrease in the rate of *comK* transcription and that these rates resemble that when both *spo0A* and *rok* are inactivated, suggests strongly that the two proteins work together in repressing *comK* transcription.

**Figure 7:**
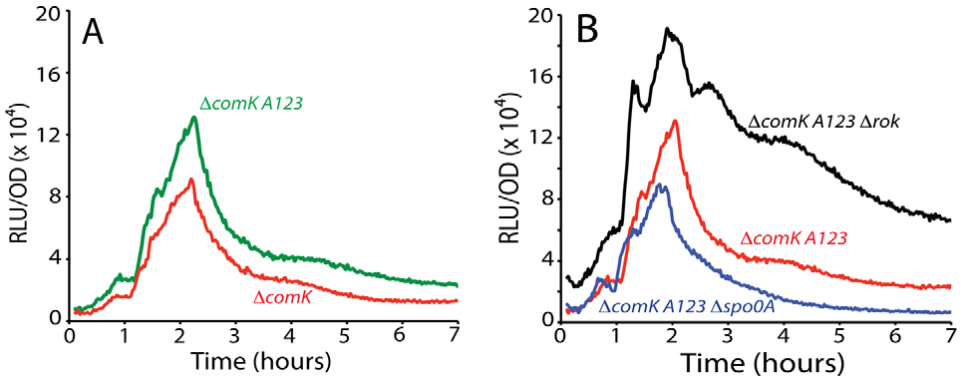
Effect of combined mutation of *A1*, *2*, and *3* on P*comK* expression. Panel A shows the effect of this triple mutation. Panel B shows its effect in *Δrok* and *Δspo0A* backgrounds.

Inactivation of either *A1* or *3* reduced the uptick to the level of the *spo0A* null-mutant, in support of the hypothesis that these are activating sites for interaction with 0A∼P (Figure 5C and 5D). Unexpectedly, inactivation of *A2* had the opposite effect, increasing expression dramatically, nearly to the level of the *rok* mutant (compare Figure 6A and 6B). The strain with both the Δ*rok* and *A2* mutation exhibited an amplitude equal to that of the *rok* mutation alone (compare Figure 6A and 6B). These results suggested that *A2* might overlap a binding site for Rok, and that 0A∼P may act at this sequence, competing for Rok binding or otherwise interfering with Rok activity. In fact, in the sequence covering *A2* and extending between the centers of *A1* and *3*, 44 out of 54 bases are either A or T (Figure 3), and Rok has been found to bind preferentially to AT-rich DNA [29]. When *A1*, *2* and *3* were mutated simultaneously, the maximum expression decreased by about half from the level of the *A2* mutant alone (compare Figure 6A and Figure 7A) showing that *A1* and *3* retained an activating role even when *A2* was inactivated. When *rok* was eliminated and *A1*, *2* and *3* were also mutated, the amplitude of the profile increased slightly compared to the *A123* triple mutant in the *rok^+^* background (Figure 7B), much less than the nearly five-fold increase caused by inactivation of *rok* in the wild type promoter background (Figure 1C). It is likely that our mutations have not completely eliminated Rok binding. This conclusion is supported by the demonstration that Rok binding with lowered affinity is retained nearby when A2 is inactivated (Figure 4D).

When a *spo0A* knockout was introduced into the *A123* strain, a small decrease in amplitude (∼30%, compared to a nine-fold decrease with the wild type promoter (Figure 1B)) was observed (Figure 7B), supporting the conclusion that the major role of 0A∼P is to antagonize Rok.

### A model for uptick regulation

The working model suggested by the above data requires that both the increase and decrease of *comK* basal transcription are largely determined by a continual rise in the amount of OA∼P. We have shown that the rate of *spo0A* transcription increases and is then maintained throughout the time of the uptick, with a pattern similar to that of core promoters e.g. P*synthA*, suggesting that this continual rise does occur (Figure S6). In addition, the transcription of a *luc* fusion to the OA∼P-dependent *sdp* promoter increases throughout the uptick (not shown), confirming that the amount of OA∼P in the cell increases.

The data suggest that during the upward segment of the P*comK* uptick, 0A∼P amplifies an inherent rise in P*comK* activity by binding to *A1*, *2* and *3*, thereby interfering with the activity of Rok. Three pieces of evidence support this role of OA-P as an anti-repressor. First, the inactivation of A2, identified from its sequence as an OA∼P binding site, imparts a Rok-like phenotype. Second, A2 and its surrounding sequence is AT-rich, as expected for a Rok binding site and third, in a Δ*rok* background, the Δ*spo0A* phenotype is essentially lost.

The inherent rise that is amplified by 0A∼P is a general one, reflecting some aspect of cell physiology that occurs as growth slows. In addition to its activity as an anti-repressor of Rok, there must be another, relatively minor component of 0A∼P activation. This is apparent from a comparison of the uptick in a Δ*comK* Δ*rok* Δ*spo0A* strain with a Δ*comK* Δ*rok spo0A^+^* strain (Figure 1C). This minor component may be due to interference with the activity of CodY, which plays a relatively small role in repression at P*comK* [28], [32] (and results not shown). It is also possible that 0A∼P acts as a classical activator, contacting RNAPol.

The decrease in P*synthA* expression begins at the same time as in P*comK*, but is less abrupt and possesses a prominent shoulder (compare Figure 1B with Figure 2B). When *rok* alone or both *spo0A* and *rok* were inactivated, a similar shoulder was observed in the decline of P*comK* expression (Figure 1C, Figure 2B) and the same was true with the *R1* and *R2* mutants (Figure 5A and 5B). The data suggest that increasing concentrations of 0A∼P interact with *R1* and *R2*, reducing expression and that repression by Rok aids in shutting down P*comK* expression.

### The concentration of 0A∼P sets limits on competence

Our working model suggests that the uptick will occur within an intermediate range of 0A∼P concentration. As a test of this prediction, we wished to measure the fluorescence intensities in individual cells of a *comK^+^* strain co-expressing fluorescent protein fusions to promoters that report respectively 0A∼P concentration and P*comK* activity. To interpret these data, it was first necessary to determine the level of P*comK* expression above which cells can be classified as competent. We therefore carried out a calibration experiment using a P*comK-cfp* fusion co-expressed with the ComK-dependent promoter P*comG*, fused to *yfp*, collecting data at 1.5 and 4 hours after the onset of stationary phase (Figure S7). Before 1.5 hours, the fluorescent signals are too weak to permit accurate data collection. The average expression of both markers increased between the two time points. In Figure S7B, most of the cells cluster with low levels of both YFP and CFP. Those with higher amounts of P*comG-yfp* expression have more than 50 units of CFP fluorescence. These cells comprise 13.3% of the total population, in good agreement with the range of values (10–20%) reported in the literature for the fraction of cells that become competent [8], [11], [20], [33]. This value was transferred to Figure S7A, in which a box is shown surrounding cells which fall within the upper 13.3% of CFP fluorescence. It is satisfying that the lower edge of this box includes all of the cells with elevated expression of *comK-cfp*, showing that the transitions had reached a limit at the earlier time, as expected [8], [11]. This lower limit of P*comK-cfp* expression at the earlier time point corresponded to a value (in arbitrary units) of 36. This value was then used for further experiments, to determine which cells had expressed sufficient *comK* at 1.5 hours after the onset of stationary phase to eventually transition to competence. (For comparison, the background signal due to auto-fluorescence in cells not encoding *cfp* is 12–16). One other feature of Figure S7 is noteworthy. Although the high *cfp* values tend to occur in cells with high *yfp* values, there is considerable scatter. At a given level of P*comK-cfp* expression, many values for P*comG* expression are possible, and *vice versa*. This may reflect the time of transition, so that cells that became competent earlier have more YFP. There may also be significant noise in the expression of P*comG*.

**Figure 8:**
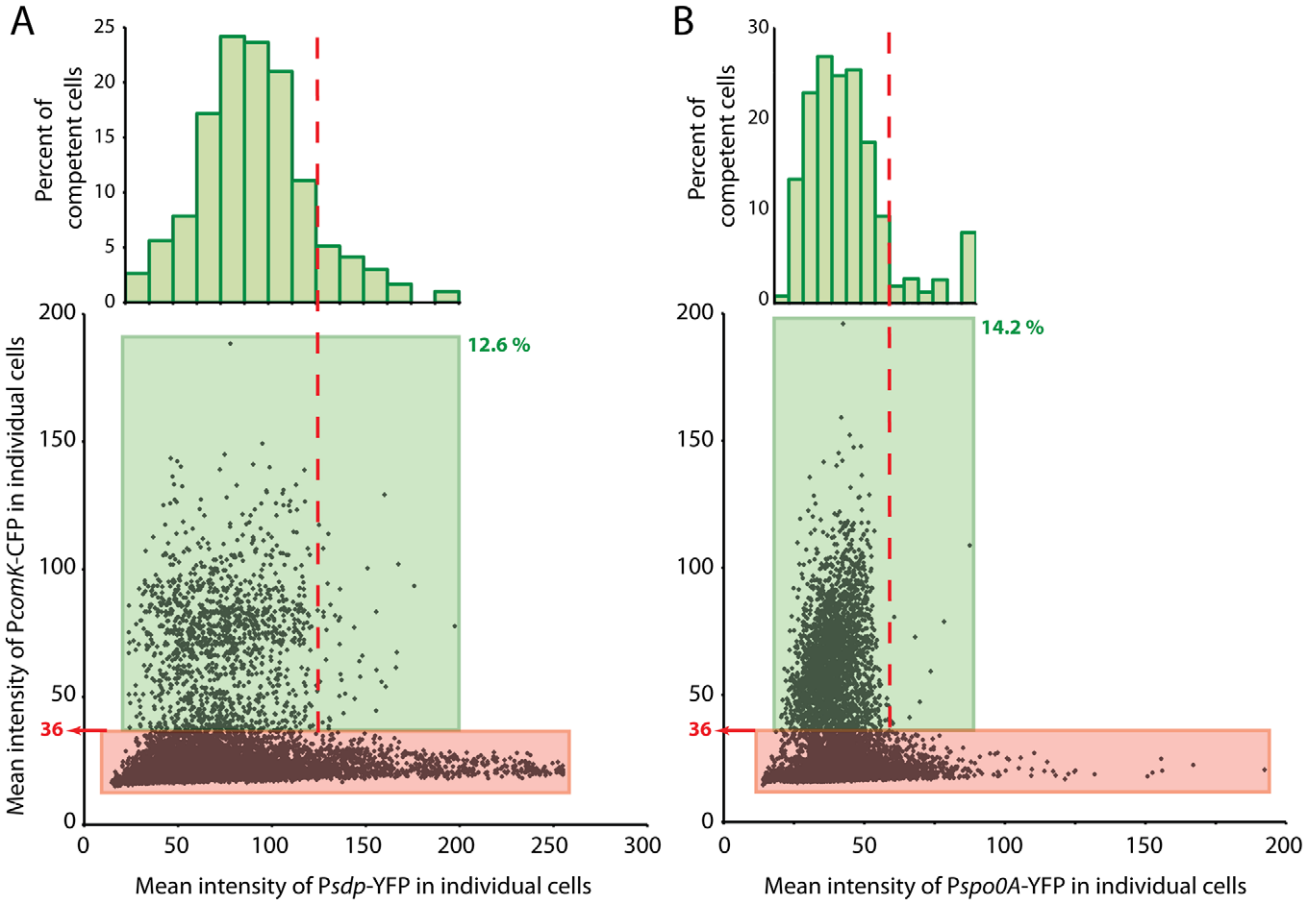
Co-expression of P*comK-cfp*. With P*sdpA-yfp* (panel A) or with P*spo0A-yfp* (panel B). Cells were segmented microscopically and the average pixel intensities in the CFP and YFP channels were recorded for each cell. The green boxes surround competence-expressing cells, with the lower limit of the boxes set from the threshold value (36) derived from Figure 8. These cells comprise 12.6% of the 11,238 cells measured for panel A and 14.2% of the 14,369 cells measured for panel B. The numbers of cells with CFP values in excess of the threshold were deposited into bins and displayed as a histogram, normalized to the total number of cells within each bin of YFP values. These data have been plotted on a histogram as a percent of total competent cells, with similar results (Figure S7).

*sdp* encodes a toxin, required for cannibalism, that is produced by cells that possess low to intermediate levels of OA∼P [34]. P*sdp* is activated by 0A∼P and in a population of cells provides a sensitive reporter of the average concentration of this molecule [35]. Figure 8A presents data from cells that co-express P*comK-cfp* and P*sdp*-*yfp*. These data were collected 1.5 hours after the onset of stationary phase and may therefore be compared with the calibration results in Figure S7A. In Figure 8A, a green box encloses cells that exceed the threshold value of 36 for P*comK-cfp* and these were found to comprise 12.6% of the total population. This value is close to the value of 13.3% determined in Figure S7A. Inspection of the scatter plot in Figure 8A suggests that above a YFP fluorescence level approximated by the vertical dashed line there are very few competent cells. In panel A there are a total of 11,238 cells represented, of which 1,419 have been classified as competent (within the green box). Of these cells, 48 are to the right of the dashed line, and these comprise 2.9% of all the cells to the right of this line. The equivalent percentage of competent cells with P*sdp* expression lower than this limit (to the left of the dashed line) is 14.4. These proportions differ with P<0.001, based on a two-tailed t-test. This decrease in the probability of transitions with high 0A∼P is predicted by the model for the 0A∼P dependence of P*comK* expression presented above. In this experiment, two types of cells may possess a high level of YFP without transitioning to competence. Some may have experienced a rapid rise in the concentration of 0A∼P, which reached an inhibitory level before P*comK* could be expressed. Others may have experienced a gradual increase in 0A∼P but simply by chance did not transition to high P*comK* expression. The histogram in panel A shows the distribution of competent cell numbers, as a percent of the total number of cells within each “slice” of YFP fluorescence. This distribution confirms that competence transitions occur within an intermediate range of P*sdp* expression and therefore within an intermediate concentration range of 0A∼P. It also suggests that there is an optimal 0A∼P concentration that maximizes the probability of competence.

Based on the following argument, it was of interest to compare these results with an analogous experiment in which P*comK-cfp* was co-expressed with P*spo0A-yfp* (Figure 8B). *A priori*, it is not known whether P*spo0A-yfp* expression would accurately reflect the level of 0A∼P in the cell because of two uncertainties. First, at a given time all the Spo0A may not be phosphorylated. Second, if the cell-to-cell variation in transcription of *spo0A* were dominated by intrinsic noise, there would be little correlation between the YFP fluorescence of a given cell derived from P*spo0A-yfp* and the amount of Spo0A protein, encoded by the wild type *spo0A* gene [36]. Comparison of the two panels of Figure 8 shed some light on these uncertainties and therefore has implications for the kinetics of Spo0A phosphorylation and the nature of noise in the amount of this molecule. As in panel A, the data in panel B shows a paucity of competent cells above a level of YFP approximated by the vertical dashed line. A calculation comparable to the one described above confirms that this paucity is highly significant (P<0.001). This comparison strongly suggests that the variation from cell-to-cell in the rate of expression from P*spo0A-yfp* is dominated by extrinsic noise, so that expression from the wild type and P*spo0A-yfp* promoters in each cell is correlated. If there were a major contribution from intrinsic noise in the firing of P*spo0A*, there would be little relationship between the expression from P*spo0A* and from P*comK* and the points in panel B would therefore extend over the entire range of P*spo0A-yfp* values. We have observed that the pattern of luciferase expression from P*spo0A* is quite similar to that of P*synthA* (Figure S5), suggesting that a global change in the physiology of these transition state cells may be responsible for changes in the average amount of *spo0A* expression and perhaps also for extrinsic noise in expression from P*spo0A*. A relative unimportance of intrinsic noise would be expected if Spo0A were abundant in these cells [37] and in fact it has been estimated that there are about 2000 molecules of this protein per cell during growth [38]. Further, the similarity in the patterns of P*comK-cfp* expression in the two panels, including in the respective histograms, is consistent with the notion that the phosphorelay is finely tuned to rapidly phosphorylate newly synthesized Spo0A as it becomes available.

As a whole, these results support the conclusion that 0A∼P plays both positive and negative roles in determining the basal level of *comK* expression and hence the probability of transitions to competence. As noted above, the histograms shown in Figure 8A and 8B suggest that there is an optimum level of 0A∼P for competence. However, at each level of 0A∼P, most cells do not transition to competence and there must be another source of noise that determines whether a given cell becomes competent. We will return to this point below.

### Stochastic simulations of the uptick model

To test the plausibility of OA∼P's dual role as a positive and negative regulator of the *comK* promoter, we simulated the network with Gillespie's stochastic simulation algorithm [39] with our primary output being expression from the *comK* promoter over time. Since our objective was to verify that Rok and Spo0A alone could define an uptick in *comK* expression, we only incorporated the binding sites for Spo0A and Rok described above. In addition, we developed a simple model to account for the global decline in transcription following entry into the stationary phase of the growth cycle (see Text S1 for details of the model, including all parameter values (Table S3) and the reactions (Table S4)).

**Figure 9:**
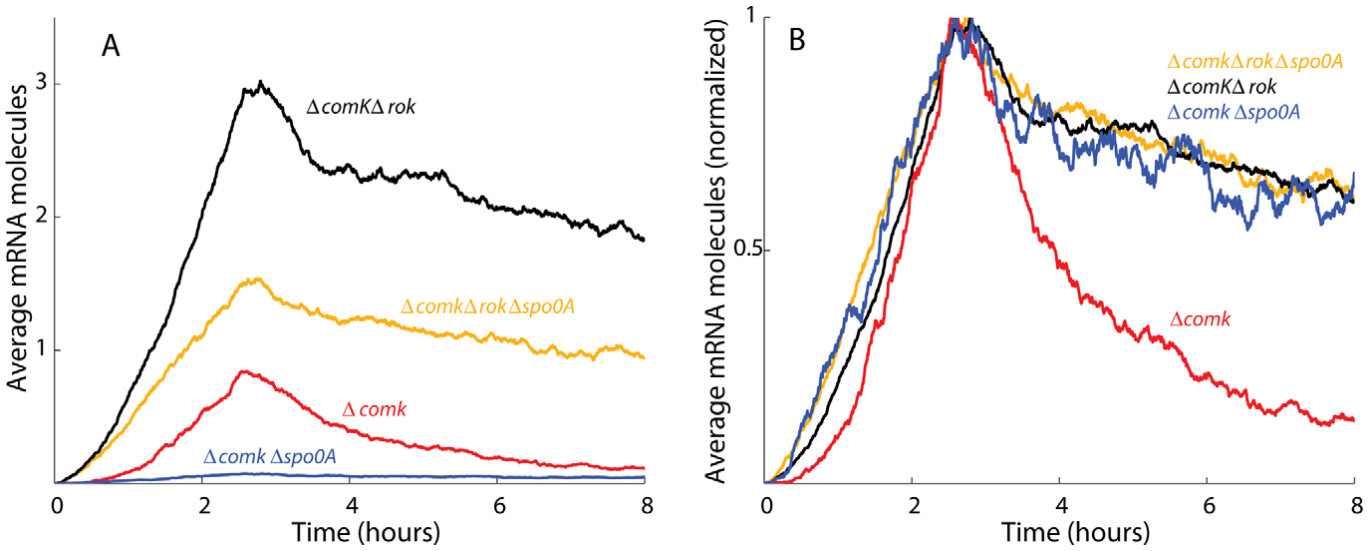
Stochastic simulation data match the experimentally observed *comK* expression. (A) The average *comK* output of 5000 Gillespie simulations reproduces the relative amplitudes of the uptick in *ΔcomK*, *ΔcomKΔrok*, *ΔcomKΔrokΔspo0A* and *ΔcomKΔspo0A* mutants (compare to Figure 1C). (B) The curves are normalized to a peak amplitude of 1 and plotted on the same axes to emphasize the sharp downturn in expression in the simulated Δ*comK* strain (compare to Figure S5). The details of the simulation are presented in Text S1.

With the appropriate parameters, we found that our model captured the temporal dynamics of the *comK* promoter and qualitatively captured the relative amplitudes of the various null-mutant strains (Figure 9A, analog to Figure 1C). By normalizing the amplitudes of the uptick in each strain, one can see the sharpened decay in the Δ*comK* Δ*rok* strain (Figure 9B, analog of Figure S5).

Our simulations suggest the possibility of an additional role of OA∼P as a classical activator that recruits RNA polymerase to the promoter. If OA∼P only activated *comK* expression as an antirepressor of Rok, then the promoter with OA∼P bound at sites A123 would have the same probability of recruiting polymerase as the bare promoter without any Rok. In our simulation output, however, we observed that the Δ*comK* Δ*rok* and the Δ*comK* Δ*rok* Δ*spo0A* strains yielded identical expression of *comK* unless OA∼P enhanced the transcription rate when bound at sites A123. Given that null-mutant strains of other regulatory factors of competence showed no major change in the uptick amplitude when measured by the luciferase assay, our simulation favors the additional role of OA∼P as a direct activator of *comK*.

Using our simulations, we addressed the question of whether fluctuations in OA could transmit to fluctuations in the expression of *comK*, thus potentially determining which cells become competent and which do not. We incorporated two independent *comK* promoters with identical binding affinities and observed that the number of transcripts from the two promoters was largely uncorrelated (the correlation coefficient ranged from 0.06 to 0.22) at various time points in the simulation (Table S5). This supports the view that variability in Spo0A is not a major determinant in whether or not a cell becomes competent, but rather that *comK* noise is the major factor as suggested previously [8]. Instead, the concentration of 0A∼P serves only to determine the window by shaping the probability of whether or not cells will become competent, without explicitly dictating cell fate.

## Discussion

### The uptick mechanism

**Figure 10:**
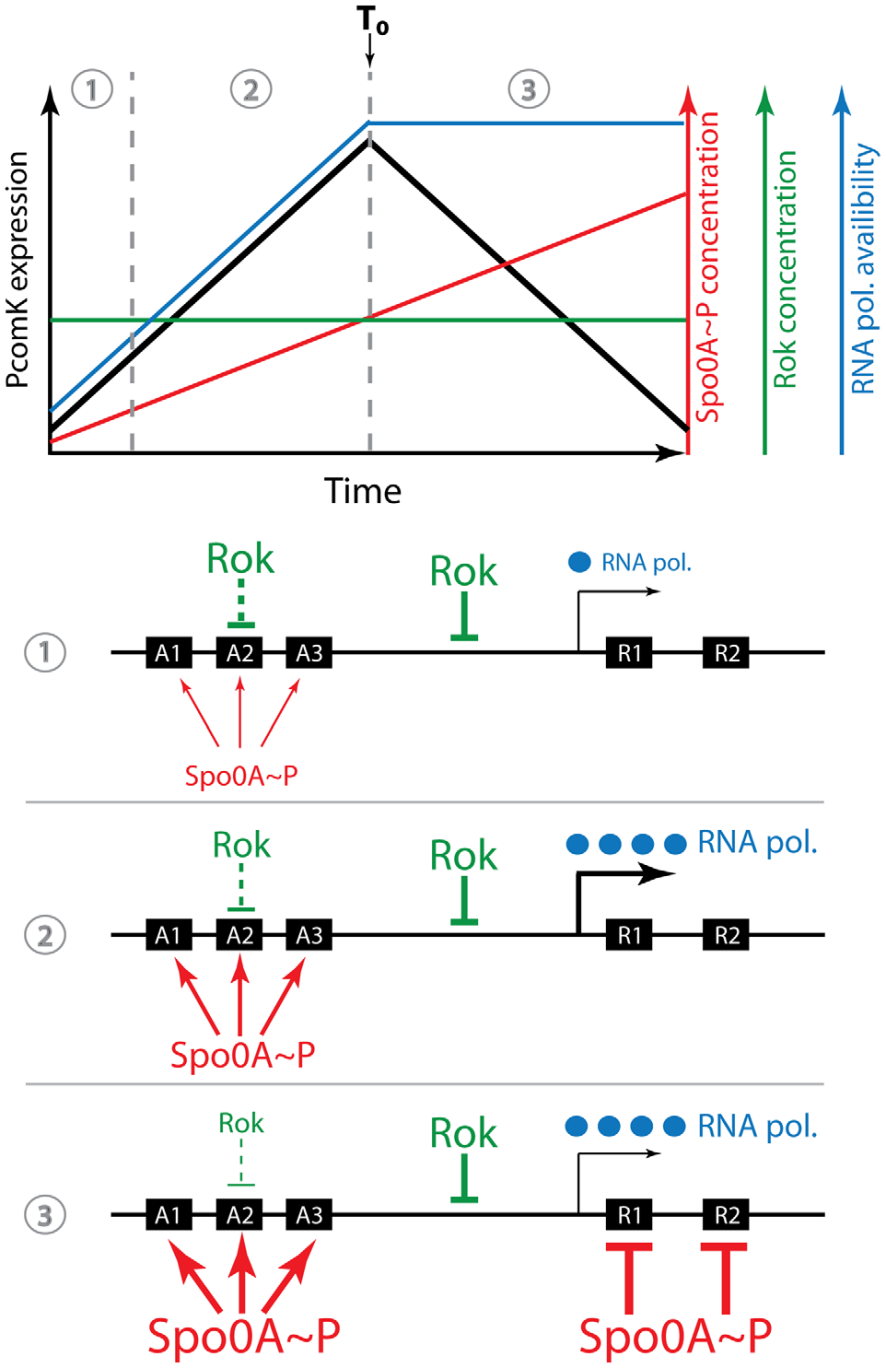
Cartoon of the uptick mechanism. The top portion of the figure shows a graphical representation of the Rok and OA∼P concentrations, as well as the availability of RNAPol and the rate of *comK* basal transcription during the transition to stationary phase. (RNAPol availability is used as a plausible stand-in for the cause of the global increase in transcription we have observed). The peak rate of transcription coincides with T_0_, the time of departure from exponential growth. When the concentrations of available RNAPol and of OA∼P are low (1) Rok is dominant and the rate of *comK* transcription is also low. As the concentration of OA∼P increases further, Rok is antagonized at sites A1, A2 and A3 and at the same time RNAPol becomes more available. As a result the rate of *comK* transcription increases (2). Finally, the OA∼P concentration reaches a level that is able to repress at R1 and R2 and *comK* transcription slows (3). In reality, of course, three demarcated periods of time do not exist. Note that the concentration of Rok remains constant throughout and both RNAPol and OA∼P work to counteract its effects. Rok works at an unidentified site in addition to A1–3, shown here between A3 and R1. For simplicity, the availability of RNAPol is shown as constant after T_0_, although our data would suggest that it varies somewhat.

The probability that a cell will enter the competent state increases and then decreases as a culture departs from exponential growth. The present work identifies two temporally variable factors that determine this transient uptick (Figure 10). First, we have discovered a previously unsuspected increase in the basal firing rate of the P*comK* promoter as cells approach stationary phase, which seems to be characteristic of core promoter elements in general, even one that depends on a minor sigma factor. This increase must be due to factors extrinsic to the “core” promoters themselves and may reflect the metabolic state of the cell, e.g. the content of nucleotide pools, the conformational state of the chromosome or the release of RNAPol from the transcription of stable RNA as growth slows [23]. In the cartoon representation of Figure 10, we have arbitrarily represented this extrinsic factor as an increase in available RNAPol. In any event, the extrinsic change may serve to couple competence regulation to cellular physiology, particularly to the growth rate, so that as growth slows, the probability of transitions to competence would increase. It is possible that a similar increase in the rate of *spo0A* transcription (Figure S6) also contributes to other forms of Spo0A-dependent stationary phase expression.

The second variable factor is 0A∼P, which binds to *A1*, *2* and *3*, amplifying the increase in P*comK* activity that is due to the core promoter activity, acting principally by antagonizing the repressing effect of Rok. During the rising portion of the uptick an increasing concentration of 0A∼P antagonizes Rok (and perhaps CodY as well) (Figure 10). In randomly selected cells, this causes the concentration of ComK to approach the threshold for transition to competence, raising the global probability that the promoter will fire. P*comK* activity decreases more abruptly than that of the core promoter (Figure 2B), because as the concentration of 0A∼P rises, it begins to occupy *R1* and *R2*. Rok contributes to this decrease because it acts as a repressor, helping OA∼P shut off transcription. It is likely that Rok does this by acting at a site in addition to A2, as represented in Figure 10, because the downturn in the *A2* mutant strain is more abrupt than in the Δ*rok A2* double mutant (Figure 6B). Thus, 0A∼P allows transitions to occur, but also closes the window of opportunity. Rok modulates this process as a repressor by working against 0A∼P during the upward part of the uptick and by accelerating the down turn, but does not itself vary in amount (Figure S3). In this model, the behavior of the core promoter and the increase in the concentration of 0A∼P are the primary variable factors that determine the timing of the uptick profile.

Stochastic simulations of the model described here successfully reproduced the experimental behavior of the system, including the relative amplitudes and the temporal dynamics of *comK* basal transcription in the wild type and mutant strains. Furthermore the modeling assumption, based on experimental evidence, that OA∼P and Rok assist one another in effecting repression at P*comK*, successfully reproduced the dynamics of repression in single and double *rok spo0A* mutants (Figure 1C, Figure 9, and Figure S5).

Although the model described above is plausible, we cannot be certain about the individual roles of A1, A2 and A3 and it is prudent to consider alternative possibilities. For example, because inactivation of A2 results in an increase in the amplitude of the uptick, it is conceivable that A2 is a repression site for OA∼P binding. As noted above, this is unlikely because inactivation of *rok* in the A2 mutant background has little effect (Figure 6A and 6B), suggesting strongly that Rok and OA∼P work on the same pathway. A similar conclusion can be drawn from comparison of single and double mutants in *rok* and *spo0A* (Figure 2). Although the inactivation of *rok* alone increases the amplitude of the uptick, further deletion of *spo0A* fails to reduce this amplitude to the level of the *spo0A* single mutant. This also suggests that Rok and OA∼P work on the same pathway. Another complex and unlikely possibility is that OA∼P recruits Rok for binding to A2, explaining why inactivation of either A2 or Rok have the same effect on the uptick. However, Figure 4D shows that Rok can bind to A2 with high affinity in the absence of OA∼P.

### An emerging theme in *Bacillus* development

The regulation of the *comK* uptick now joins two other cases of timed regulation dependent on 0A∼P, in which this molecule acts positively and then negatively as its average concentration increases. Spo0A acts to cause a burst in the expression of *sinI*, a key regulator in the early development of biofilms, which is expressed bimodally [15], [31]. The regulation of *sinI* has been explained by differential affinity of 0A∼P for activating and repressing sites [31]. The second case concerns *sdp*, an operon needed for cannibalism, which encodes a toxic extracellular factor that kills non-producing cells with less 0A∼P, permitting the producing cells access to nutrients and thereby delaying their commitment to sporulation [34]. *sdp* transcription is activated and then repressed by 0A∼P [35]. However, this regulation differs from than of *comK* and *sinI*. *sdp* is repressed by AbrB and its induction is explained by the extreme sensitivity of the *abrB* promoter to repression by 0A∼P and also by the activation of *abbA* transcription. The AbbA protein binds to AbrB, further derepressing *sdp* [40]. High concentrations of 0A∼P, on the other hand, repress *sdp* transcription by direct binding. Thus, *comK*, *sinI* and *sdp* are all turned on by 0A∼P at low concentrations and turned off as the amount of 0A∼P increases, with this versatile protein acting by various combinations of activation, repression and anti-repression. *comK* is the only known target gene for which 0A∼P acts as an anti-repressor. Competence thus joins sporulation, cannibalism and biofilm formation as subject to temporal regulation by Spo0A∼P. As *B. subtilis* enters its developmental pathways, it uses the gradual accumulation of 0A∼P as a clock, to orchestrate the timing of gene expression.

### The source of noise for competence transitions

Figure 8A shows that competence develops within an intermediate range of P*sdp-yfp* concentrations. The few cells with elevated CFP at the low end of the YFP distribution is consistent with the observation that the uptick is amplified by Spo0A∼P, although the number of cells in this region of the scatter plot is too low to establish the statistical significance of this observation. Nevertheless, it is known that *spo0A* mutant cells are not competent [24] and these cells are likely to be below the threshold for competence transitions. Cells at the high end of the YFP distribution likewise have a low probability of being competent because their transition windows have closed. This upper limit is due to repression exerted at *R1* and *R2*, as shown here. Within the intermediate range of YFP concentrations, corresponding to an intermediate range of Spo0A∼P, conditions are permissive for transitions to occur. Clearly at each concentration of Spo0A∼P within this permissive range, indicated by expression of P*sdp-yfp*, a wide range of ComK-CFP concentrations can exist and most cells fail to become competent (Figure 8A). Likewise, at each level of P*comK-cfp* expression above the threshold value for competence, a range of YFP values are possible. It follows from these results that cell-to-cell variation in the content of Spo0A∼P alone does not determine cell fate.

In agreement with this conclusion, our previous evidence suggested that the major source of noise for fate determination is the firing rate of P*comK* [8]. The data in Figure 8 are consistent with that result, as is the uptick model that we advanced above. Thus, we suggest that the noise in transcription from P*comK* determines cell fate and that 0A∼P amplifies P*comK* expression, permitting about 10–20% of the cells to exceed the transition threshold for amplification of *comK* expression by auto-induction. 0A∼P then shuts down transcription when it reaches higher concentrations, driving the transition rate toward zero. Our earlier experiments suggested that the P*comK* noise is intrinsic, because the numbers of transcripts derived from two *comK* promoters in the same cell were poorly correlated at the peak of the uptick [8], when there is about one *comK* transcript in the average cell [8] and therefore the average number of ComK protein molecules is likely to be low. Under these conditions we would expect intrinsic noise in *comK* expression to dominate cell-to-cell variation [37] and to be the major factor selecting cells for the competent fate. The simulation results described above support this conclusion, shown by the poor correlation observed when the outputs of independent *comK* promoters were compared (Table S5). Although noise in *comK* is the major determinant, 0A∼P acts as a temporal gate, modulating the probability that random fluctuations in *comK* transcription will lead to competence.

### A more general role for OA∼P

It has been well established that a gradual increase in the concentration of OA∼P governs development in *B. subtilis*. Although competence was known to require *spo0A*, the role of OA∼P in this pathway was imperfectly understood. It now appears that the probability of transition to the competent state is determined by the programmed increase in OA∼P, as are the development of biofilms, cannibalism and spores. OA∼P thus plays a general role in the temporal control of development and competence must be subject to the complex signaling that governs the synthesis of this molecule, involving multiple kinases, phosphatases and extracellular peptides. Despite this commonality, competence differs from sporulation, cannibalism and biofilm formation in an important respect. Asynchrony during the gradual post-exponential increase in OA∼P concentration is most likely the major determinant of which cell is selected for these last three fates. In contrast, it is noise in *comK*, a dedicated gene, that governs the choice of cells for competence, although OA∼P helps to set the temporal window of opportunity.

## Materials and Methods

### Microbiological methods

*Bacillus subtilis* strains were constructed by transformation into BD630 (*his leu met*) with selection for the appropriate antibiotic resistance marker, and all comparisons were with isogenic strains. For transformation, competent cultures were prepared and incubated in competence medium with transforming DNA (∼1 µg/ml) for 30 min at 37°C [24]. The strains are listed in Table S1. All the growth experiments were carried out in competence medium [24].

### Luciferase assay

For the detection of luciferase activity, strains were first grown in LB medium to an optical density at 600 nm (OD_600_) of 2. Cells were then centrifuged and resuspended in fresh competence medium, adjusting all the cultures to an OD_600_ of 2. These pre-cultures were then diluted 20 fold in fresh competence medium and 200 µl was distributed in each of two wells in a 96-well black plate (Corning). 10 µl of luciferin was added to each well to reach a final concentration of 1.5 mg/ml (4.7 mM). The cultures were incubated at 37°C with agitation in a PerkinElmer Envision 2104 Multilabel Reader equipped with an enhanced sensitivity photomultiplier for luminometry. The temperature of the clear plastic lid was maintained at 38°C to avoid condensation. Relative Luminescence Units (RLU) and OD_600_ were measured at 1.5 min intervals. The data were plotted as RLU/OD versus time from the beginning of growth.

### Construction of luciferase promoter fusion strains

A 1 Kb fragment ending with the initiating codon of the gene of interest, and containing the promoter, was amplified by PCR from the *B. subtilis* chromosome. A single nucleotide was inserted in the primer to restore the correct reading frame. Primers are listed in Table S2 (PcomK1 and 2). The PCR fragment was cut by *Kpn*I/*Nco*I in sites present at the extremities of the primers used for the amplification. In parallel, the luciferase gene was cut from plasmid pGL3 (Promega) by *Nco*I/*Bam*H1 digestion. A three-fragment ligation was then carried out between the promoter of interest, the luciferase gene and plasmid pUC18Cm digested with *Kpn*I and *Bam*H1. The resulting plasmid, pCU18cm-*promoter::luc*, which cannot replicate autonomously in *B. subtilis* was used to transform *B. subtilis* with selection for chloramphenicol resistance, where it integrated by single crossover. This event reconstructed the “normal” regulatory region in front of the fusion and a complete copy of the gene of interest, downstream of the fusion.

When necessary, mutations were incorporated in the promoter in front of *luc* using the ‘Change-It Multiple Mutation Site Directed Mutagenesis’ kit (USB). The primers used in these site-directed mutagenesis constructions are listed in Table S2. The resulting plasmids were verified by sequencing and then integrated by Campbell-like recombination and the structure of the integration event was verified by sequencing a relevant PCR fragment from the chromosome.

### Construction of YFP promoter fusion strains

A 1 Kb fragment ending with the initiating codon of the gene of interest, and containing the promoter, was amplified by PCR from the *B. subtilis* chromosome. Primers are listed in Table S2 (Pspo0A3, Pspo0A4, Psdp1 and Psdp2). The PCR fragment was cut by *EcoRI*/*XhoI* at sites present at the extremities of the primers used for the amplification. In parallel, the *yfp* gene was amplified using the primers *yfp8* and *yfp9* (see Table S2). A three-fragment ligation was then carried out between the promoter of interest, the *yfp* gene and plasmid pUC18Cm digested with *EcoR*I and *Bam*H1. The resulting plasmid, pCU18cm-*promoter::yfp*, which cannot replicate autonomously in *B. subtilis*, was used to transform *B. subtilis* with selection for chloramphenicol resistance, where it integrated by single crossover. This event reconstructed the “normal” regulatory region in front of the fusion and a complete copy of the gene of interest, downstream of the fusion.

### Construction of Sigma A and Sigma H core promoter fusion strains

The *luc* gene was amplified from the plasmid pGL3 (Promega) using the primers ‘sigAcore’ or ‘sigHcore’ and ‘luc2’ (see Table S2). The ‘sigAcore’ or ‘sigHcore’ primers allowed us to add the core promoters and the ribosomal binding site of *spoVG* upstream of the *luc* gene. The PCR products were cut using *Bam*H*1* and *Eco*RI and cloned in the plasmid pDR111 digested with the same enzymes. The resulting plasmids, pDR111-P*sigAcore*-*luc* or pDR111-P*sigHcore*-*luc*, were used to transform *B. subtilis* where they integrated by double crossover at the *amyE* locus.

### Construction of a *comK* deletion

To inactivate *comK*, we replaced it cleanly with a tetracycline resistance cassette without using a vector. We first amplified 1 kb fragments upstream and downstream of the gene. These fragments are each flanked with one restriction site at the junction with respectively the ‘start’ or the ‘stop’ of the gene. In parallel, we amplified the *tet* cassette flanked with the same two restriction sites. The three fragments were then digested and ligated together and the ligated DNA was purified through a QIAquick column. The desired product, corresponding to ligation of the three fragments, was purified from an agarose gel. The purified band was then amplified by PCR using the outside primers previously used to amplify the upstream and downstream fragments. After further purification on a QIAquick column, the full fragment (flanking sequences+antibiotic cassette) was used to transform *B. subtilis* with selection for tet-resistance yielding a double crossover event between the chromosome and the region of homology and replacing the gene with the antibiotic cassette.

### Purification and phosphorylation of 0A∼P and purification of Rok

Spo0A was purified following a published protocol [41] as modified [42]. Non-radioactive phosphoramidate was synthesized [43] and used to phosphorylate the Spo0A. On a Superdex 200 column the resulting Spo0A appeared to be predominantly in the dimer form. Rok was purified as a C-terminal His6 fusion as previously described [26].

### Gel mobility shift assays

The primers used to amplify the probe DNA fragments by PCR (OAwt, OA123, ORwt and OR1&2) are listed in Table S2. The 5′ ends of these probe fragments were labeled using [g-^32^P]-ATP and T4 polynucleotide kinase. For this, about 500 ng of DNA was incubated in 50 µl final volume, with 10 units of enzyme and with 40 µCi of [g-^32^P]-ATP (specific activity of 4500 Ci/mmol). For the gel shift experiments, a typical assay mixture contained (in a final volume of 20 µl), 10 mM Tris-HCl, pH 8.0, 50 mM NaCl, 1 mM EDTA, 1 mM dithiothreitol (DTT), 5% (v/v) glycerol, 0.5 µg of bovine serum albumin, approximately 0.5 ng of probe and the indicated concentrations of 0A∼P protein. After 5 min of incubation at 37°C, 10 µl of this mixture was loaded onto a native 5% (w/v) polyacrylamide TBE Ready Gel (Bio-Rad) and electrophoresed in 0.5× TBE buffer for 1 h at 100 V cm^−1^. The gels were pre-run for about an hour. Radioactive fragments were detected by autoradiography. Gel shifts with Rok were performed as described previously [26].

### Microscopy

From *B. subtilis* cultures growing in the plate reader in competence medium, samples were taken at 1.5 or 4 hours after the entrance to stationary phase. Aliquots (200 µl) of each sample were centrifuged and resuspended in 100 µl of PBS. One µl of each sample was placed on a 1% agarose pad. All images were acquired using Volocity v 5.1 (Perkin Elmer) and a Nikon 90i fluorescence microscope with filters appropriate for detection of CFP and YFP. Segmentation and measurements of pixel intensities were carried out with the Volocity measurement tools.

## Supporting Information

Figure S1 Reproducibility of the luciferase assay. Four replicate wells inoculated with the Δ*comK* P*comK-luc* strain were grown in competence medium in the plate reader. Light output (left ordinate) and OD_600_ were measured. Each color represents one well. The reproducible inflection point at 2.25 hours (T_0_, vertical arrow) is regarded as the point of transition to stationary phase. The groups of replicate curves are connected to their Y-axes by black arrows.
(TIF)

Figure S2 Effects of phosphorelay mutations on the P*spo0A* uptick. The P*spo0A-luc* construct was combined with the indicated null-mutations and luciferase activity was measured. Individual knockouts of *kinA* and *kinB* had small but reproducible effects on this activity (not shown). Single knockouts of *kinC*, *kinD* and *kinE* had negligible effects if any (not shown). There is clearly considerable redundancy in the contributions of these kinases under the conditions used, although KinA and KinB play the major roles. The residual activity in the *spo0F* mutant is presumably due to direct phosphorylation of Spo0A by KinA and B.
(TIF)

Figure S3 Western blotting for Rok. Samples were taken from a wild type and a *spo0A* null mutant at hourly intervals beginning one hour before the transition to stationary phase (T_-1_). The cells were lysed, electrophoresed by SDS-PAGE and blotted for reaction with anti-Rok antiserum. Equal amounts of total protein were loaded on each lane.
(TIF)

Figure S4 Sequences of the synthetic SigA and SigH dependent promoters (A) and expression of the P*synthH-luc* promoter fusion (B). P*synthH* is expressed at a low rate under these conditions and the light output trace is consequently noisy.
(TIF)

Figure S5 Effects of various mutations on the declining portion of the P*spo0A* uptick. The peak values of the uptick curves for the *R1*, *R2*, P*synthA* and wild type promoters and of the curves in the Δ*rok* and Δ*rok* Δ*spo0A* backgrounds were all normalized to 100%.
(TIF)

Figure S6 Expression of P*spo0A-luc*. The construct was introduced by Campbell-like recombination, placing the *luc* coding sequence under control of both the vegetative and sporulation promoters of *spo0A* with all their upstream regulatory sequences. Compare to Figure 2B (P*synthA* expression) and Figure S4 (P*synthH* expression). Because this curve represents a rate, the total amount of Spo0A protein is expected to continuously rise.
(TIF)

Figure S7 Co-expression of P*comK-cfp* with P*comG-yfp* at 1.5 hours (panel A) and 4 hours (B) after the onset of stationary phase. Cells were segmented and the average pixel intensities in the CFP and YFP channels were recorded for each cell. The green box in panel B surrounds competence-expressing cells, which comprise 13.3% of the total. The box in panel A was drawn to also enclose the upper 13.3% of the CFP distribution. Panel A includes data from 9,722 cells and panel B from 12,223 cells. The lower limit of the green box in panel A was used to derive a threshold value for P*comK-cfp* expression (36 arbitrary fluorescence units).
(TIF)

Table S1 Bacterial strains used for this study.
(PDF)

Table S2 Sequence of oligonucleotides primers used.
(PDF)

Table S3 Rate coefficients.
(PDF)

Table S4 Reactions.
(PDF)

Table S5 Correlations.
(PDF)

Text S1 Stochastic simulations.
(PDF)
