# Supplementary figures and images for: Spo0A∼P Imposes a Temporal Gate for the Bimodal Expression of Competence in Bacillus subtilis
Source: PLoS Genet. 2012 Mar 8;8(3):e1002586. doi: 10.1371/journal.pgen.1002586 (PMC3297582; doi:10.1371/journal.pgen.1002586)

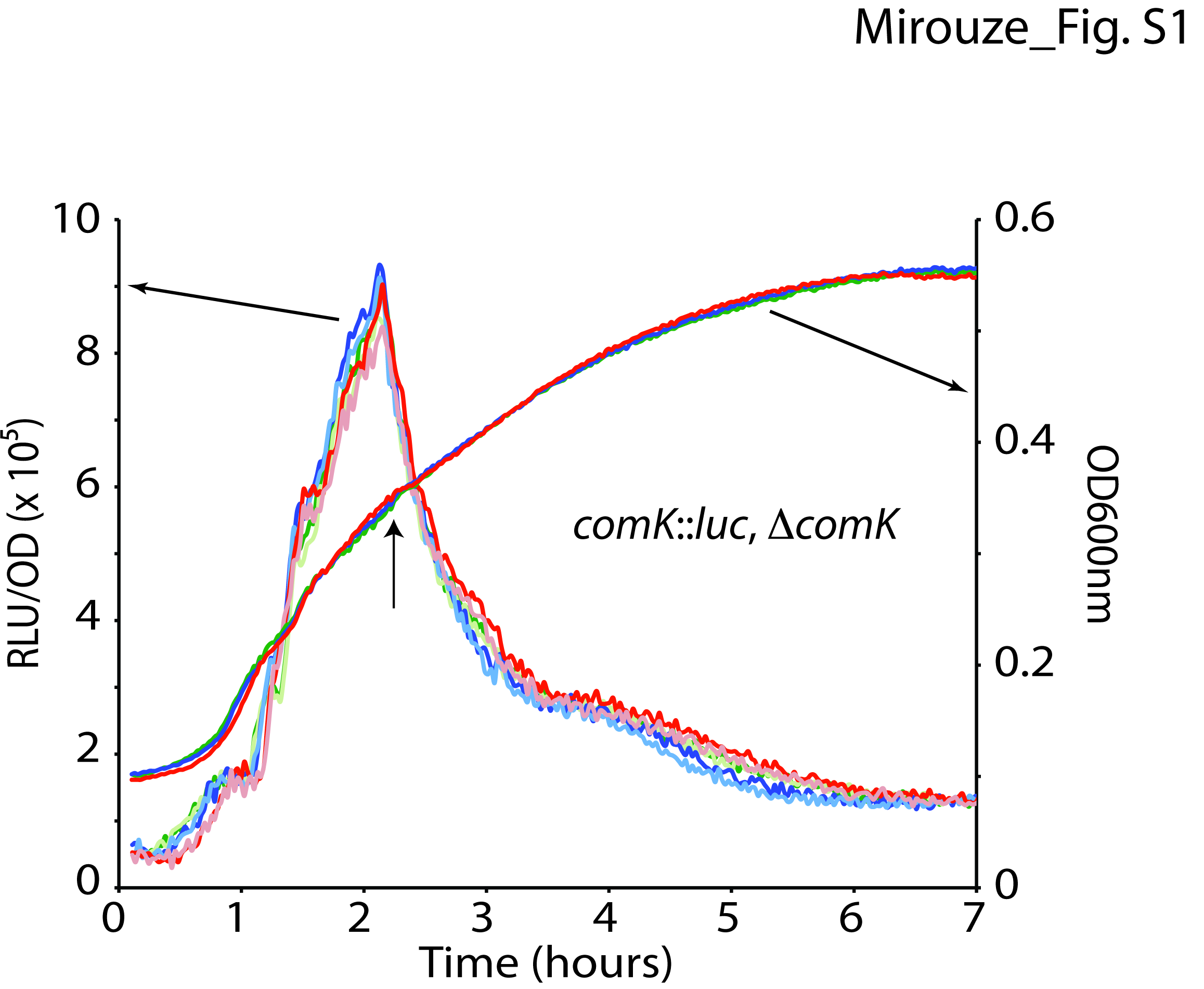

Supplement: Figure S1 — Reproducibility of the luciferase assay. Four replicate wells inoculated with the ΔcomK PcomK-luc strain were grown in competence medium in the plate reader. Light output (left ordinate) and OD600 were measured. Each color represents one well. The reproducible inflection point at 2.25 hours (T0, vertical arrow) is regarded as the point of transition to stationary phase. The groups of replicate curves are connected to their Y-axes by black arrows. (TIF) [file pgen.1002586.s001.tif]

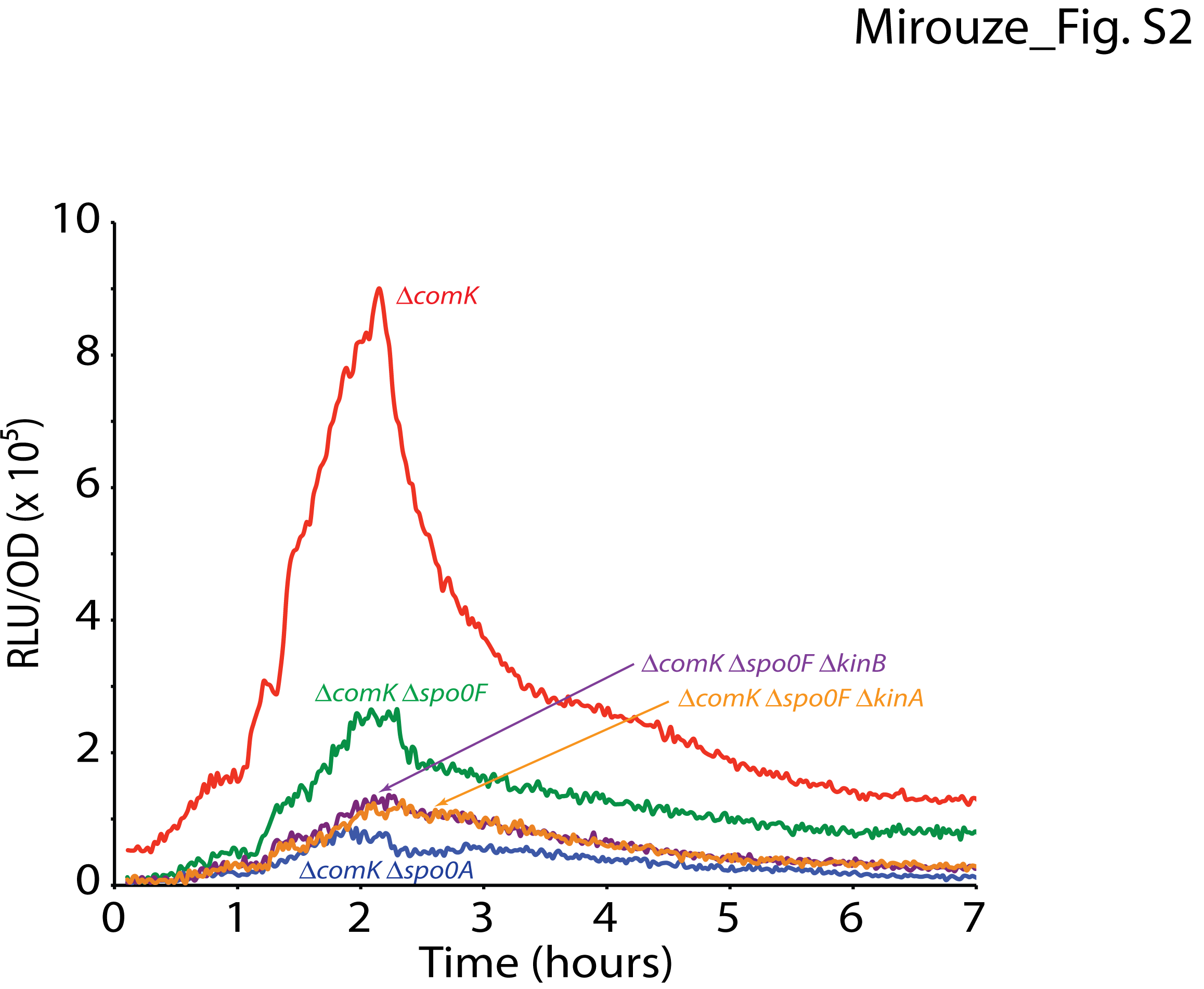

Supplement: Figure S2 — Effects of phosphorelay mutations on the Pspo0A uptick. The Pspo0A-luc construct was combined with the indicated null-mutations and luciferase activity was measured. Individual knockouts of kinA and kinB had small but reproducible effects on this activity (not shown). Single knockouts of kinC, kinD and kinE had negligible effects if any (not shown). There is clearly considerable redundancy in the contributions of these kinases under the conditions used, although KinA and KinB play the major roles. The residual activity in the spo0F mutant is presumably due to direct phosphorylation of Spo0A by KinA and B. (TIF) [file pgen.1002586.s002.tif]

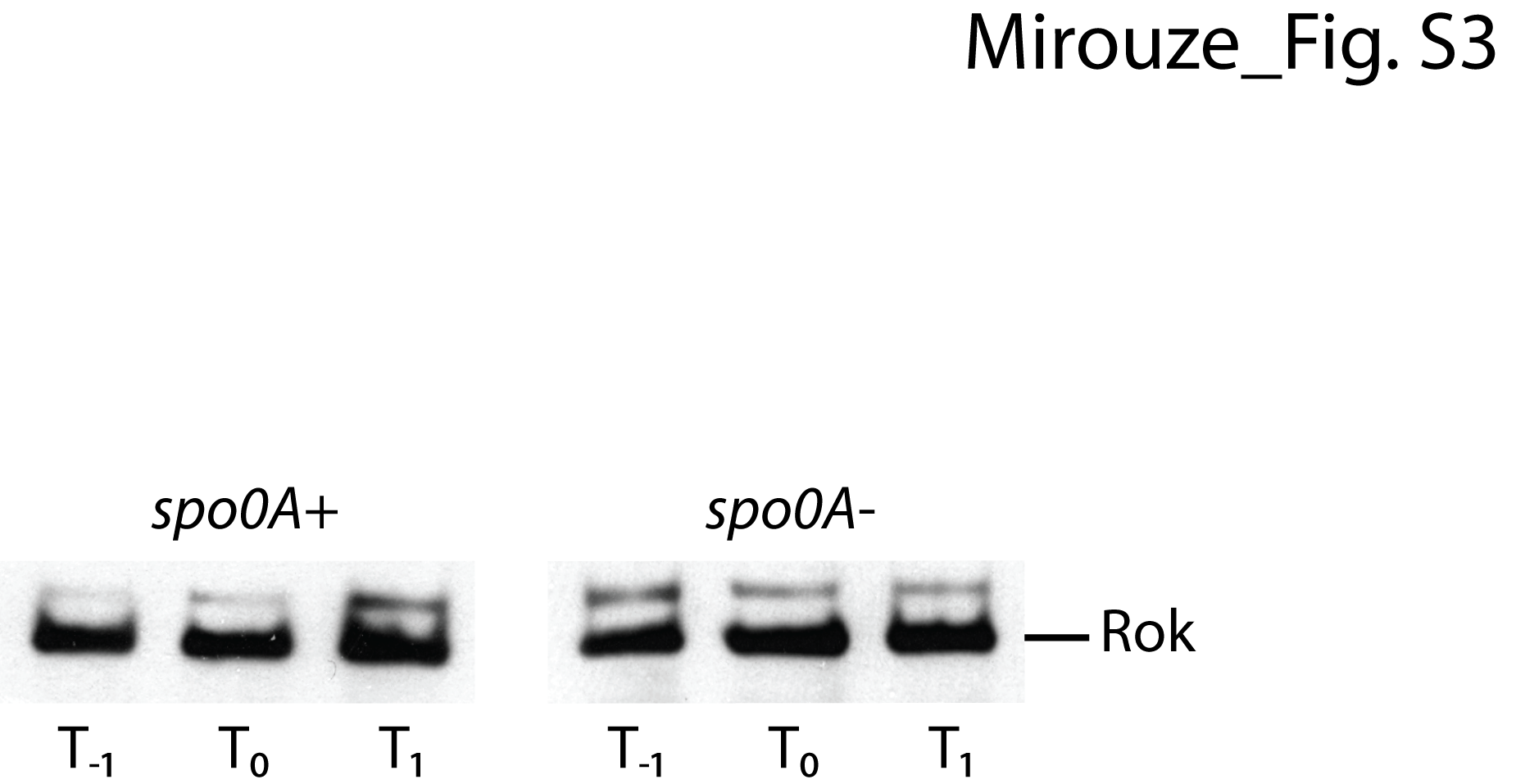

Supplement: Figure S3 — Western blotting for Rok. Samples were taken from a wild type and a spo0A null mutant at hourly intervals beginning one hour before the transition to stationary phase (T-1). The cells were lysed, electrophoresed by SDS-PAGE and blotted for reaction with anti-Rok antiserum. Equal amounts of total protein were loaded on each lane. (TIF) [file pgen.1002586.s003.tif]

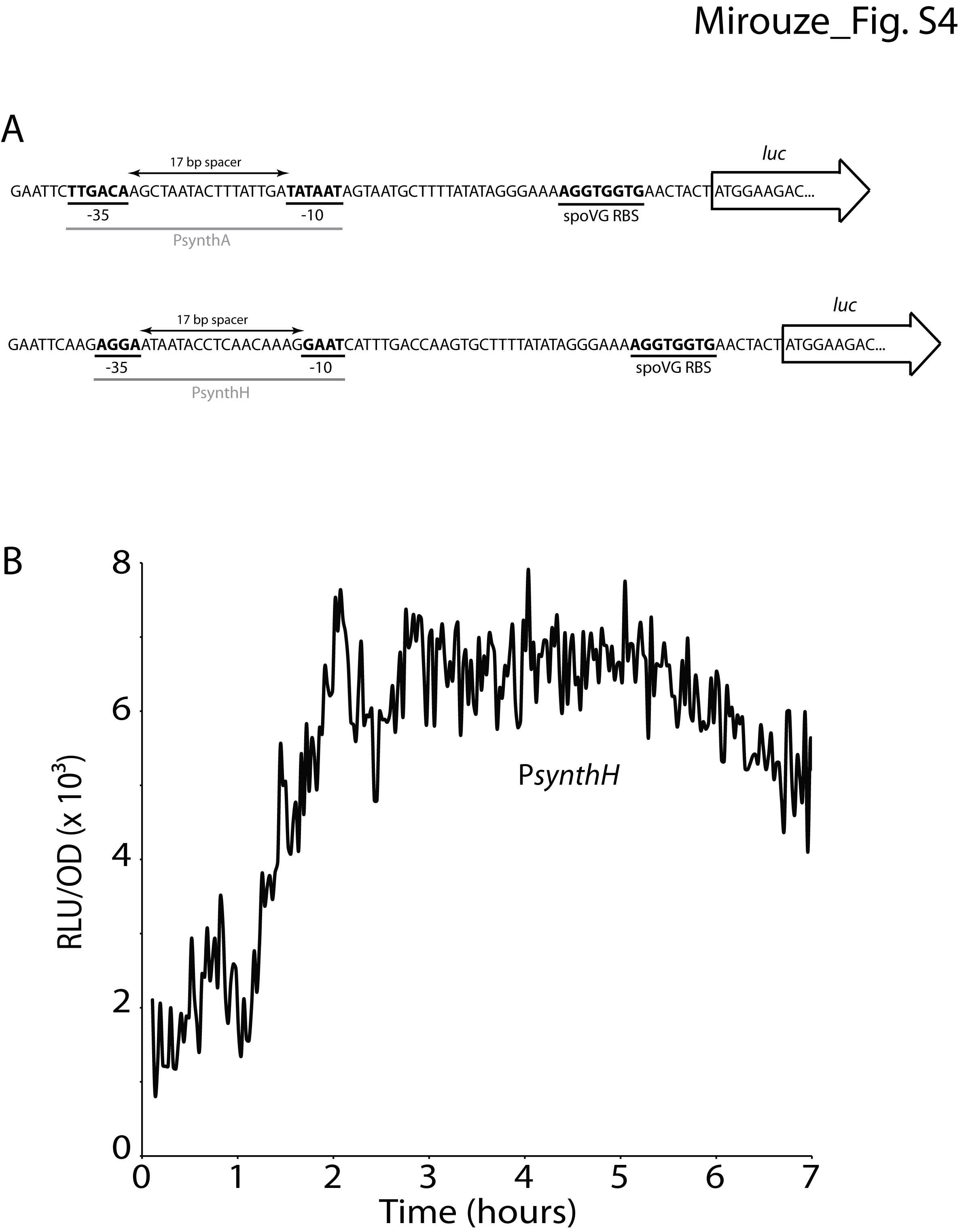

Supplement: Figure S4 — Sequences of the synthetic SigA and SigH dependent promoters (A) and expression of the PsynthH-luc promoter fusion (B). PsynthH is expressed at a low rate under these conditions and the light output trace is consequently noisy. (TIF) [file pgen.1002586.s004.tif]

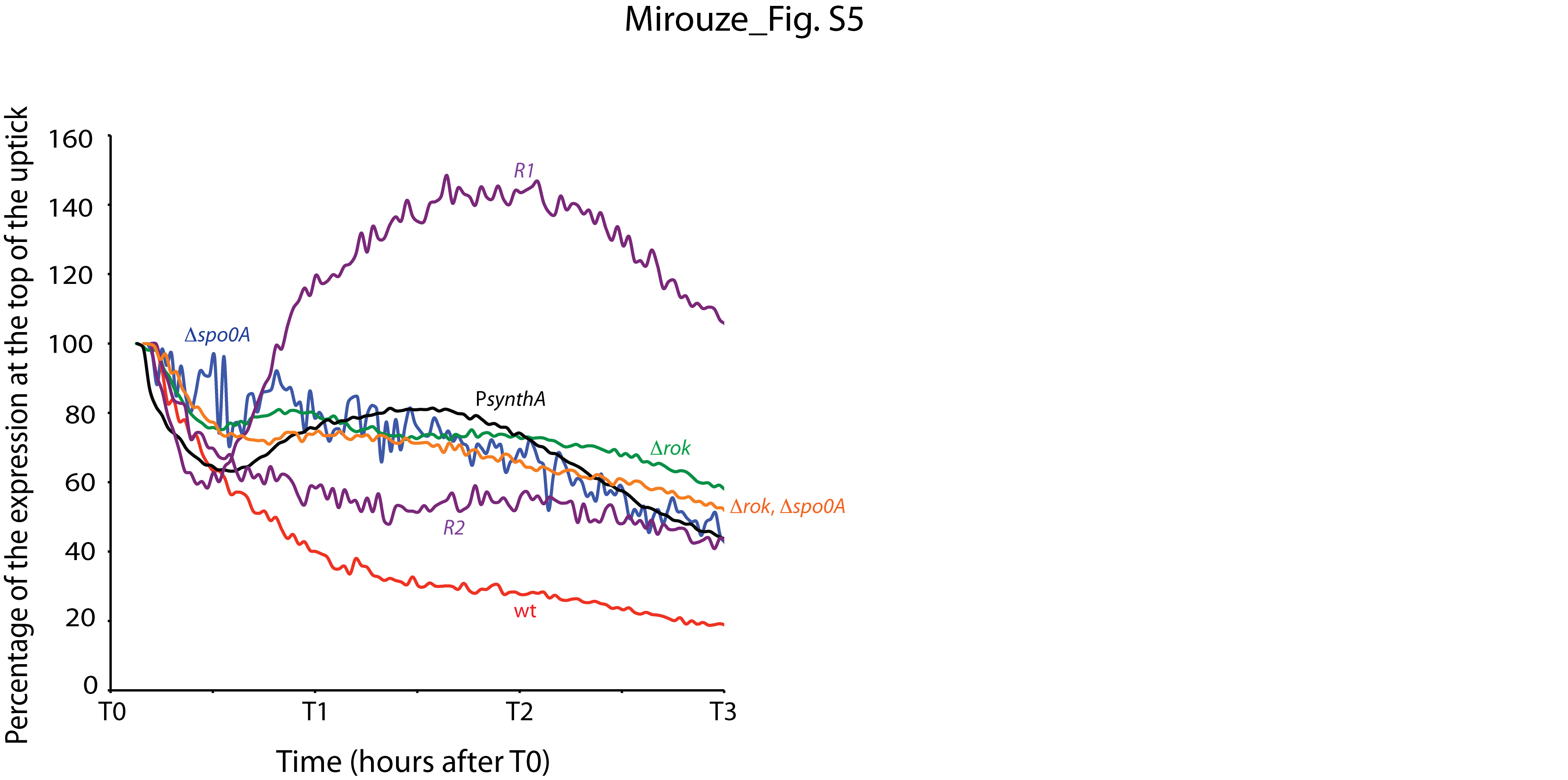

Supplement: Figure S5 — Effects of various mutations on the declining portion of the Pspo0A uptick. The peak values of the uptick curves for the R1, R2, PsynthA and wild type promoters and of the curves in the Δrok and Δrok Δspo0A backgrounds were all normalized to 100%. (TIF) [file pgen.1002586.s005.tif]

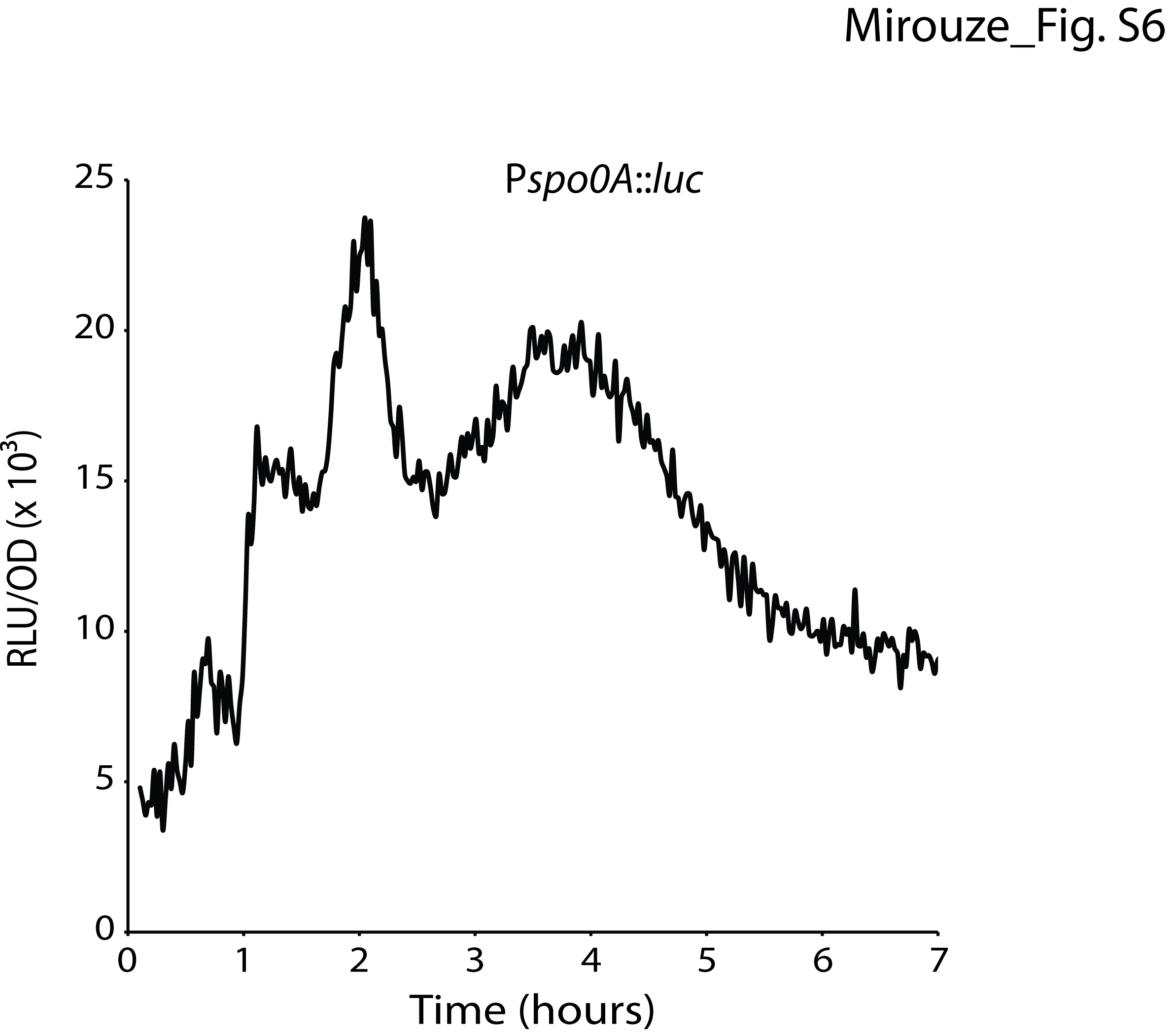

Supplement: Figure S6 — Expression of Pspo0A-luc. The construct was introduced by Campbell-like recombination, placing the luc coding sequence under control of both the vegetative and sporulation promoters of spo0A with all their upstream regulatory sequences. Compare to Figure 2B (PsynthA expression) and Figure S4 (PsynthH expression). Because this curve represents a rate, the total amount of Spo0A protein is expected to continuously rise. (TIF) [file pgen.1002586.s006.tif]

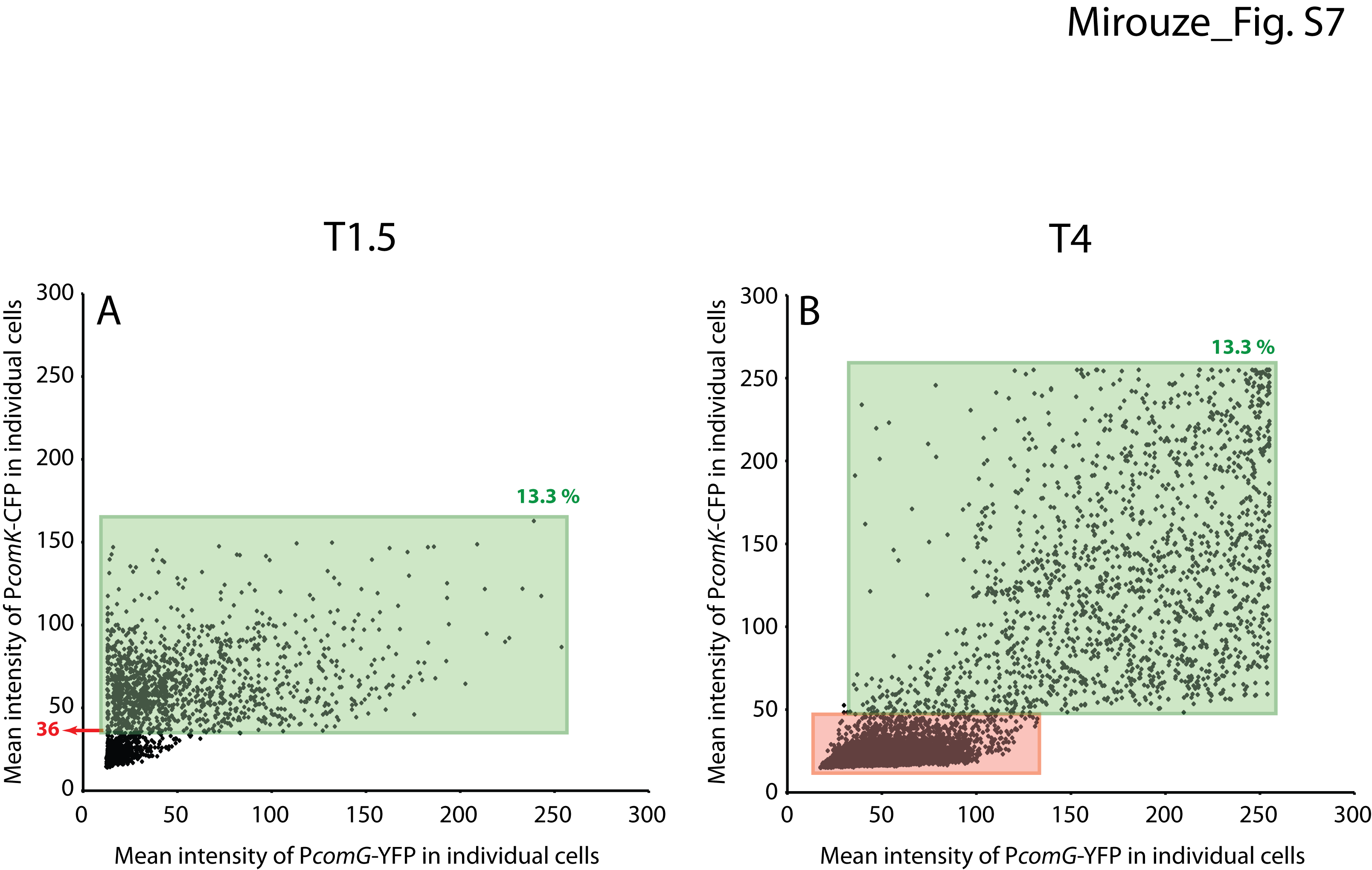

Supplement: Figure S7 — Co-expression of PcomK-cfp with PcomG-yfp at 1.5 hours (panel A) and 4 hours (B) after the onset of stationary phase. Cells were segmented and the average pixel intensities in the CFP and YFP channels were recorded for each cell. The green box in panel B surrounds competence-expressing cells, which comprise 13.3% of the total. The box in panel A was drawn to also enclose the upper 13.3% of the CFP distribution. Panel A includes data from 9,722 cells and panel B from 12,223 cells. The lower limit of the green box in panel A was used to derive a threshold value for PcomK-cfp expression (36 arbitrary fluorescence units). (TIF) [file pgen.1002586.s007.tif]
