# Supplementary material for: Spo0A∼P Imposes a Temporal Gate for the Bimodal Expression of Competence in Bacillus subtilis
Source: PLoS Genet. 2012 Mar 8;8(3):e1002586. doi: 10.1371/journal.pgen.1002586 (PMC3297582; doi:10.1371/journal.pgen.1002586)
Supplement: Table S1 — Bacterial strains used for this study. (PDF) [file pgen.1002586.s008.pdf]

Spo0A~P imposes a temporal gate for the bimodal expression of competence in *B. subtilis*

Table S1

Table S1: Bacterial strains used for this study

B. subtilis **strains**	**Genotype[1]**	**Source / reference[2]**
BD630	*his leu-8 metB5,*	Lab strain
BD4773	*his leu-8 metB5,* P*comK*::*luc cm*	This study
BD4893	*his leu-8 metB5,* P*comK*::*luc cm,* Δ*comK spc*	This study
BD4954	*his leu-8 metB5,* P*comK*::*luc cm,* Δ*comK spc*, Δspo0F *ΔPstI*	This study
BD4954	*his leu-8 metB5,* P*comK*::*luc cm,* Δ*comK spc*, Δspo0F *ΔPstI*, ΔkinB *phleo*	This study
BD4954	*his leu-8 metB5,* P*comK*::*luc cm,* Δ*comK spc*, Δspo0F *ΔPstI*, ΔkinA *ery*	This study
BD5025	*his leu-8 metB5,* P*comK*::*luc cm,* Δ*comK tet*	This study
BD5699	*his leu-8 metB5,* P*comKA1*::*luc cm,* Δ*comK tet*	This study
BD5700	*his leu-8 metB5,* P*comKA2*::*luc cm,* Δ*comK tet*	This study
BD5701	*his leu-8 metB5,* P*comKA3*::*luc cm,* Δ*comK tet*	This study
BD5702	*his leu-8 metB5,* P*comKR1*::*luc cm,* Δ*comK tet*	This study
BD5703	*his leu-8 metB5,* P*comKR2*::*luc cm,* Δ*comK tet*	This study
BD5731	*his leu-8 metB5,* P*comKA2*::*luc cm,* Δ*comK tet*, Δ*rok spc*	This study
BD5773	*his leu-8 metB5,* P*comK*-CFP *kan*, P*spo0A*-YFP *cm*	This study
BD5784	*his leu-8 metB5,* P*comK*::*luc cm,* Δ*comK tet*, Δ*spo0A kan*	This study
BD5785	*his leu-8 metB5,* P*comK*::*luc cm,* Δ*comK tet*, Δ*rok spc*	This study
BD5786	*his leu-8 metB5,* P*comK*::*luc cm,* Δ*comK tet*, Δ*rok spc*, Δ*spo0A kan*	This study
BD5837	*his leu-8 metB5,* P*comKA123*::*luc cm,* Δ*comK tet*	This study
BD5840	*his leu-8 metB5,* P*comKA123*::*luc cm,* Δ*comK tet*, Δ*spo0A kan*	This study
BD5845	*his leu-8 metB5,* P*comK*-CFP *kan*, P*comGA*-YFP *cm*	This study
BD5848	*his leu-8 metB5,* P*comKA123*::*luc cm,* Δ*comK tet*, Δ*rok spc*	This study
BD5922	*his leu-8 metB5,* P*sigH*::*luc spc*	This study
BD5923	*his leu-8 metB5,* P*sigA*::*luc spc*	This study
BD5945	*his leu-8 metB5,* P*comK*-CFP *kan*, P*sdp*-YFP *cm*	This study
PP530	*his leu-8 metB5,* P*spo0A*::*luc cm*	[1]

[1] The P*comK*::*luc* fusion was integrated at the native *comK* locus by single reciprocal (Campbell-like) recombination, preserving the wild-type native locus and placing the fusion under control of all the upstream regulatory sequences.

The P*sigA*::*luc* and P*sigH*::*luc* fusions were integrated at the *amyE* locus by double cross-over. The correct insertion at the *amyE* locus was verified by the loss of the amylase activity.

The P*spo0A*-YPF and P*sdp*-YFP fusions were integrated at their native locus by single reciprocal (Campbell-like) recombination, preserving the wild-type native locus and placing the fusion under control of all the upstream regulatory sequences.

[2] The sources of the knockouts mutations, other than Δ*comK tet*, are as follows: *Δcomk spc* [2], Δ*spo0A* (M. Fujita), Δ*kinA* [3], Δ*kinB* [3], Δ*spo0F* (I. Smith), Δ*rok* [4]. The source of the P*comK*-CFP fusion is [5].

References

1. Mirouze N, Prepiak P, Dubnau D (2011) Fluctuations in spo0A transcription control rare developmental transitions in Bacillus subtilis. PLoS Genet 7: e1002048.
2. Hamoen LW, Smits WK, de Jong A, Holsappel S, Kuipers OP (2002) Improving the predictive value of the competence transcription factor (ComK) binding site in *Bacillus subtilis* using a genomic approach. Nucleic Acids Res 30: 5517-5528.
3. Quisel JD, Burkholder WF, Grossman AD (2001) In vivo effects of sporulation kinases on mutant Spo0A proteins in Bacillus subtilis. J Bacteriol 183: 6573-6578.
4. Albano M, Smits WK, Ho LT, Kraigher B, Mandic-Mulec I, et al. (2005) The Rok protein of Bacillus subtilis represses genes for cell surface and extracellular functions. J Bacteriol 187: 2010-2019.
5. Maamar H, Raj A, Dubnau D (2007) Noise in gene expression determines cell fate in Bacillus subtilis. Science 317: 526-529.
