# Supplementary material for: Spo0A∼P Imposes a Temporal Gate for the Bimodal Expression of Competence in Bacillus subtilis
Source: PLoS Genet. 2012 Mar 8;8(3):e1002586. doi: 10.1371/journal.pgen.1002586 (PMC3297582; doi:10.1371/journal.pgen.1002586)
Supplement: Table S2 — Sequence of oligonucleotides primers used. (PDF) [file pgen.1002586.s009.pdf]

Spo0A~P imposes a temporal gate for the bimodal expression of competence in *B. subtilis*

Table S2

Table S2: Sequence of oligonucleotide primers used

Primer	Sequence
PcomK1	5'- GCA GCA **GGT ACC** AAA TCA CCA GCC ACC AGC TGA TCA GAA -3'
PcomK2	5'- G CAG CA**C CAT GGC** TAA AGG TGC GTC TGT TTT CTG ACT CAT ATT ATG GCC -3'
comKK0 F5	5'- GCA GCA **GGT ACC** CCA GCC ACC AGC TGA TCA GAA CCT TCC AAA CAA AC-3'
comKKO F3	5'- TGC TGC **GGA TCC** GGC CTC CAT CCT TTT TCT GCA AAA TTT ATA C-3'
comKKO R5	5'- GCA GCA **AAG CTT** TAG AAA AAT AGG AAG GAG CTG ACC GAA CAG GGC AGC TCC-3'
comKKO R3	5'- TGC TGC **TCT AGA** CTC AGG GGT ACA CAT ACG AAT GTA CAC CAA ACA G -3'
tet 5 BamHI	5'- GCA GCA **GGA TCC** CGT AGT AAG CGT GGA ACA AAG AAC GAA GAG AG-3'
tet 3 HindIII	5'- TGC TGC **AAG CTT** CGT ATT CCT CTT ATA TTA GAA TTC CTG TTA TAA AAA AAG GAT CAA TTT TGA ACT CTC TCC C-3'
comKA1	5'- CTT TTT TAT AGT ATA TGG ATA ACG GT**A AC**A AAA ATC AAT AAA TTT TTA TAA TTT TTA GA -3'
comKA2	5'- GAA AAA ATC AAT AAA TTT TTA TAA TTT T**G**A **AC**C **C**AC TAA TAC TTG GCA ATC TAT CGA CA -3'
comKA3	5'- TTT AGA CAA CTA ATA CTT GGC AAT CTA T**AA CCC** TAT CCT GCA AAA TGC CGT AAA CCG GC -3'
comKR1	5'- GAT TTT ATC TTA AAT GTT AAA AAA AC**T** TG**A** C**A**T TTT ACA AAA ACA GAT GAT AGA TTA TT -3'
comKR2	5'- AGA TGA TAG ATT ATT AGT ATA AAT TTT **C**CA **CT**A AAA GGA TGG AGG CCA TAA TAT GAG TC -3'
yfp8	5'- CGA CGA **GTC GAC CTC GAG** ATG AGT AAA GGA GAA GAA CTT TTC ACT GGA GTT GTC C -3'
yfp9	5'- CGA CGA **GGA TCC** TTA TTT GTA TAG TTC ATC CAT GCC ATG TGT AAT CCC AGC -3'
Pspo0A3	5'- CGA CGA **GAA TTC** TAA ACA GAA AAT CAA AAC GAA GCT GAT CCC AGA AAA GG -3'
Pspo0A4	5'- GCA GCA **CTC GAG** CAC GTT TCT TCC TCC CCA AAT GTA GTT AAC AGG ATT CA -3'
Psdp1	5'- GCA GCA **GAA TTC** GTG TAA ACG CTG ATT TAC CCG CAT TGA CGT GTG -3'
Psdp2	5'- GCA GCA **CTC GAG** CAT AGT AGT TCC TCC TTT TTT GAT GTA GAT TAC CTC CTC TAA GCT AAT ATA ATC ATT TCA AAA AG -3'
PsigAcore	5'- CGA CGA **GAA TTC** **TTG ACA** AGC TAA TAC TTT AT**T G**AT ATA ATA GTA ATG CTT TTA TAT AGG GAA AAG GTG GTG AAC TAC T**AT G**GA AGA CGC CAA AAA CAT AAA GAA AGG CCC G -3'
PsigAcore	5'- CGA CGA **GAA TTC** AAG **AGG A**AT AAT ACC TCA ACA AAG **GAA T**CA TTT GAC CAA GTG CTT TTA TAT AGG GAA AAG GTG GTG AAC TAC T**AT G**GA AGA CGC CAA AAA CAT AAA GAA AGG CCC G -3'
luc2	5'- GCA GCA **GGA TCC** TTA CAC GGC GAT CTT TCC GCC CTT CTT GGC CTT TA -3'
