# Supplementary material for: Spo0A∼P Imposes a Temporal Gate for the Bimodal Expression of Competence in Bacillus subtilis
Source: PLoS Genet. 2012 Mar 8;8(3):e1002586. doi: 10.1371/journal.pgen.1002586 (PMC3297582; doi:10.1371/journal.pgen.1002586)
Supplement: Table S3 — Rate coefficients. (PDF) [file pgen.1002586.s010.pdf]

Spo0A~P imposes a temporal gate for the bimodal expression of competence in B. subtilis

Table S3 Rate Coefficients

**Rate Parameter**	**Value**	**Description**
μ_{GF1}	2 $molecules^{-1} hours^{-1}$	Production rate of GF from 0 to 2.5 hours
μ_{GF2}	0 $molecules^{-1} hours^{-1}$	Production rate of GF from 2.5 to 3 hours
μ_{GF3}	0 $molecules^{-1} hours^{-1}$	Production rate of GF from 3 to 8 hours
δ_{GF1}	0 $molecules^{-1} hours^{-1}$	Degradation rate of GF from 0 to 2.5 hours
δ_{GF2}	0.5 $molecules^{-1} hours^{-1}$	Degradation rate of GF from 2.5 to 3 hours
δ_{GF3}	0.05 $molecules^{-1} hours^{-1}$	Degradation rate of GF from 3 to 8 hours
μ_o	0.5 $molecules^{-1} hours^{-1}$	Production rate of Spo0A
δ_o	0.2 $molecules^{-1} hours^{-1}$	Degradation rate of Spo0A
k_1	5000 $molecules^{-1} hours^{-1}$	Binding rate of Spo0A to activating site
k_2	1000 $molecules^{-1} hours^{-1}$	First dissociation rate of Spo0A from activating site
k_3	100 $molecules^{-1} hours^{-1}$	Second dissociation rate of Spo0A from activating site
k_4	10 $molecules^{-1} hours^{-1}$	Third dissociation rate of Spo0A from activating site
k_5	2500 $molecules^{-1} hours^{-1}$	Binding rate of Spo0A to repressive site (R1/R2)
k_6	2.5E6 $molecules^{-1} hours^{-1}$	First dissociation rate of Spo0A from repressive site
k_7	2500 $molecules^{-1} hours^{-1}$	Second dissociation rate of Spo0A from repressive site
k_8	1000 $molecules^{-1} hours^{-1}$	Binding rate of Rok to first repressive site (A123)
k_9	10000 $molecules^{-1} hours^{-1}$	Dissociation rate of Rok from first repressive site (A123)
k_{10}	1000 $molecules^{-1} hours^{-1}$	Binding rate of Rok to second repressive site
k_{11}	1E5 $molecules^{-1} hours^{-1}$	Dissociation rate of Rok from second repressive site when Spo0A not bound at R1/R2
k_{12}	100 $molecules^{-1} hours^{-1}$	Dissociation rate of Rok from second repressive site when Spo0A bound at R1/R2
μ_1	1 $molecules^{-1} hours^{-1}$	Transcription rate of promoter without Spo0A or Rok
μ_2	2 $molecules^{-1} hours^{-1}$	Transcription rate of activated promoter
μ_3	2E-3 $molecules^{-1} hours^{-1}$	Transcription rate of repressed promoter
δ_M	3 $molecules^{-1} hours^{-1}$	Degradation rate of *comK* transcript
