# Supplementary material for: Spo0A∼P Imposes a Temporal Gate for the Bimodal Expression of Competence in Bacillus subtilis
Source: PLoS Genet. 2012 Mar 8;8(3):e1002586. doi: 10.1371/journal.pgen.1002586 (PMC3297582; doi:10.1371/journal.pgen.1002586)
Supplement: Table S4 — Reactions. (PDF) [file pgen.1002586.s011.pdf]

Spo0A~P imposes a temporal gate for the bimodal expression of competence in B. subtilis

Table S4 Reactions

$\phi \xrightarrow{\mu_{GF1}} GF$

$\phi \xrightarrow{\mu_{GF2}} GF$

$\phi \xrightarrow{\mu_{GF3}} GF$

$GF \xrightarrow{\delta_{GF1}} \phi$

$GF \xrightarrow{\delta_{GF2}} \phi$

$GF \xrightarrow{\delta_{GF3}} \phi$

$GF + Pspo \xrightarrow{\mu_o} O + GF + Pspo$

$O \xrightarrow{\delta_o} \phi$

$P + O \xrightleftharpoons[\mathrm{k}_2]{\mathrm{k}_1} P_{O_1}$

$P_{O_1} + O \xrightleftharpoons[\mathrm{k}_3]{\mathrm{k}_1} P_{O_2}$

$P_{O_2} + O \xrightleftharpoons[\mathrm{k}_4]{\mathrm{k}_1} P_{O_3}$

$P + O \xrightleftharpoons[\mathrm{k}_6]{\mathrm{k}_5} P_{o_1}$

$P_{O_1} + O \xrightleftharpoons[\mathrm{k}_6]{\mathrm{k}_5} P_{O_1 o_1}$

$P_{O_2} + O \xrightleftharpoons[\mathrm{k}_6]{\mathrm{k}_5} P_{O_2 o_1}$

$P_{O_3} + O \xrightleftharpoons[\mathrm{k}_6]{\mathrm{k}_5} P_{O_3 o_1}$

$P_{o_1} + O \xrightleftharpoons[\mathrm{k}_7]{\mathrm{k}_5} P_{o_2}$

$P_{O_1 o_1} + O \xrightleftharpoons[\mathrm{k}_7]{\mathrm{k}_5} P_{O_1 o_2}$

$P_{O_2 o_1} + O \xrightleftharpoons[\mathrm{k}_7]{\mathrm{k}_5} P_{O_2 o_2}$

$P_{O_3 o_1} + O \xrightleftharpoons[\mathrm{k}_7]{\mathrm{k}_5} P_{O_3 o_2}$

$P + R \xrightleftharpoons[\mathrm{k}_9]{\mathrm{k}_8} P_R$

$P_{o_1} + R \xrightleftharpoons[\mathrm{k}_9]{\mathrm{k}_8} P_{o_1 R}$

$P_{o_2} + R \xrightleftharpoons[\mathrm{k}_9]{\mathrm{k}_8} P_{o_2 R}$

$P_R + O \xrightleftharpoons[\mathrm{k}_6]{\mathrm{k}_5} P_{o_1 R}$

$P_{o_1 R} + O \xrightleftharpoons[\mathrm{k}_7]{\mathrm{k}_5} P_{o_2 R}$

$P + R \xrightleftharpoons[\mathrm{k}_{11}]{\mathrm{k}_{10}} P_r$

$P_{O_1} + R \xrightleftharpoons[\mathrm{k}_{11}]{\mathrm{k}_{10}} P_{O_1 r}$

$P_{O_2} + R \xrightleftharpoons[\mathrm{k}_{11}]{\mathrm{k}_{10}} P_{O_2 r}$

$P_{O_3} + R \xrightleftharpoons[\mathrm{k}_{11}]{\mathrm{k}_{10}} P_{O_3 r}$

$P_R + R \xrightleftharpoons[\mathrm{k}_{11}]{\mathrm{k}_{10}} P_{Rr}$

$P_{o_1} + R \xrightleftharpoons[\mathrm{k}_{12}]{\mathrm{k}_{10}} P_{o_1 r}$

$P_{o_2} + R \xrightleftharpoons[\mathrm{k}_{12}]{\mathrm{k}_{10}} P_{o_2 r}$

$P_{O_1 o_1} + R \xrightleftharpoons[\mathrm{k}_{12}]{\mathrm{k}_{10}} P_{O_1 o_1 r}$

$P_{O_2 o_1} + R \xrightleftharpoons[\mathrm{k}_{12}]{\mathrm{k}_{10}} P_{O_2 o_1 r}$

$P_{O_3 o_1} + R \xrightleftharpoons[\mathrm{k}_{12}]{\mathrm{k}_{10}} P_{O_3 o_1 r}$

$P_{O_1 o_2} + R \xrightleftharpoons[\mathrm{k}_{12}]{\mathrm{k}_{10}} P_{O_1 o_2 r}$

$P_{O_2 o_2} + R \xrightleftharpoons[\mathrm{k}_{12}]{\mathrm{k}_{10}} P_{O_2 o_2 r}$

$P_{O_3 o_2} + R \xrightleftharpoons[\mathrm{k}_{12}]{\mathrm{k}_{10}} P_{O_3 o_2 r}$

$P_{o_1 R} + R \xrightleftharpoons[\mathrm{k}_{12}]{\mathrm{k}_{10}} P_{o_1 Rr}$

$P_{o_2 R} + R \xrightleftharpoons[\mathrm{k}_{12}]{\mathrm{k}_{10}} P_{o_2 Rr}$

$$P + GF \xrightarrow{\mu_1} P + GF + M$$

$$P_{O_1} + GF \xrightarrow{\mu_2} GF + M$$

$$P_{O_2} + GF \xrightarrow{\mu_2} P_{O_2} + GF + M$$

$$P_{O_3} + GF \xrightarrow{\mu_2} P_{O_3} + GF + M$$

$$P_{o_1} + GF \xrightarrow{\mu_3} P_{o_1} + GF + M$$

$$P_{o_2} + GF \xrightarrow{\mu_3} P_{o_2} + GF + M$$

$$P_{O_1o_1} + GF \xrightarrow{\mu_3} P_{O_1o_1} + GF + M$$

$$P_{O_2o_1} + GF \xrightarrow{\mu_3} P_{O_2o_1} + GF + M$$

$$P_{O_3o_1} + GF \xrightarrow{\mu_3} P_{O_3o_1} + GF + M$$

$$P_{O_1o_2} + GF \xrightarrow{\mu_3} P_{O_1o_2} + GF + M$$

$$P_{O_2o_2} + GF \xrightarrow{\mu_3} P_{O_2o_2} + GF + M$$

$$P_{O_3o_2} + GF \xrightarrow{\mu_3} P_{O_3o_2} + GF + M$$

$$P_R + GF \xrightarrow{\mu_3} P_R + GF + M$$

$$P_{o_1R} + GF \xrightarrow{\mu_3} P_{o_1R} + GF + M$$

$$P_{o_2R} + GF \xrightarrow{\mu_3} P_{o_2R} + GF + M$$

$$P_r + GF \xrightarrow{\mu_3} P_r + GF + M$$

$$P_{O_1r} + GF \xrightarrow{\mu_3} P_{O_1r} + GF + M$$

$$P_{O_2r} + GF \xrightarrow{\mu_3} P_{O_2r} + GF + M$$

$$P_{O_3r} + GF \xrightarrow{\mu_3} P_{O_3r} + GF + M$$

$$P_{Rr} + GF \xrightarrow{\mu_3} P_{Rr} + GF + M$$

$$P_{o_1r} + GF \xrightarrow{\mu_3} P_{o_1r} + GF + M$$

$$P_{o_2r} + GF \xrightarrow{\mu_3} P_{o_2r} + GF + M$$

$$P_{O_1o_1r} + GF \xrightarrow{\mu_3} P_{O_1o_1r} + GF + M$$

$$P_{O_2o_1r} + GF \xrightarrow{\mu_3} P_{O_2o_1r} + GF + M$$

$$P_{O_3o_1r} + GF \xrightarrow{\mu_3} P_{O_3o_1r} + GF + M$$

$$P_{O_1o_2r} + GF \xrightarrow{\mu_3} P_{O_1o_2r} + GF + M$$

$$P_{O_2o_2r} + GF \xrightarrow{\mu_3} P_{O_2o_2r} + GF + M$$

$$P_{O_3o_2r} + GF \xrightarrow{\mu_3} P_{O_3o_2r} + GF + M$$

$$P_{Ro_1r} + GF \xrightarrow{\mu_3} P_{Ro_1r} + GF + M$$

$$P_{Ro_2r} + GF \xrightarrow{\mu_3} P_{Ro_2r} + GF + M$$

$$M \xrightarrow{\delta_M} \phi$$
