# Supplementary material for: Spo0A∼P Imposes a Temporal Gate for the Bimodal Expression of Competence in Bacillus subtilis
Source: PLoS Genet. 2012 Mar 8;8(3):e1002586. doi: 10.1371/journal.pgen.1002586 (PMC3297582; doi:10.1371/journal.pgen.1002586)
Supplement: Table S5 — Correlations. (PDF) [file pgen.1002586.s012.pdf]

Spo0A~P imposes a temporal gate for the bimodal expression of competence in B. subtilis

Table S5 Correlations

**time (hours)**	**correlation coefficient, R**
1	0.0581
1.5	0.1282
2	0.1508
2.5	0.1780
3	0.1619
3.5	0.2169
4	0.1619
4.5	0.2094
5	0.1296
5.5	0.1376
6	0.2199
