# Supplementary material for: Spo0A∼P Imposes a Temporal Gate for the Bimodal Expression of Competence in Bacillus subtilis
Source: PLoS Genet. 2012 Mar 8;8(3):e1002586. doi: 10.1371/journal.pgen.1002586 (PMC3297582; doi:10.1371/journal.pgen.1002586)
Supplement: Text S1 — Stochastic simulations. (PDF) [file pgen.1002586.s013.pdf]

Spo0A~P imposes a temporal gate for the bimodal expression of competence in *B. subtilis*

Text S2 Stochastic simulations

We modeled *comK* expression dynamics with Gillespie's stochastic algorithm [1]. The goal of our simulation was to verify that the promoter interactions of Spo0A and Rok described in the text could define an uptick in *comK* expression. Parameters were chosen arbitrarily, although we selected the *comK* mRNA degradation rate in accordance with previously reported mRNA half-lives in *B. subtilis* [2]. We assumed, based on experimental evidence (Fig. S3), that Rok protein levels were constant throughout the simulation. We incorporated three activating sites for Spo0A (A1, A2, A3), which overlap with the repressive site of Rok. We also included two repressive binding sites (R1, R2) for Spo0A. Further, when Spo0A is bound at its repressive sites, it cooperatively recruits Rok to a second binding site distinct from A123 to strengthen repression. Since low concentrations of Spo0A activate the promoter and high concentrations repress the promoter, we included a higher Spo0A binding affinity for the A123 sites than for the R1, R2 sites (Table S3).

The global change in transcription dynamics represents a biological phenomenon independent of the molecules involved in our proposed network. We captured this general, experimentally observed trend with a species we call "global factor" (GF), a theoretical proxy that we assume scales linearly with the activity of RNA polymerase. In our model, GF was a reactant in each transcription reaction (see Supplementary table with reactions (Table S4)). We juxtaposed three Gillespie simulations, where each simulation had identical parameters except for the reactions governing the production or degradation of GF. The first segment of the simulation describes the system for the first 2.5 hours, during which GF increases linearly. The second segment describes hours 2.5-3, during which GF decreases linearly. Finally, the third segment describes hours 3-8, during which GF continues to decrease linearly, but at a slower rate than in the previous segment (Table S3). Our modified Gillespie simulation thus captured the time dependency in rate parameters necessitated by the globally observed transcription dynamics.

To test whether noise in *comK* expression was intrinsic or extrinsic, we ran the simulation with two identical promoters, and compared the mRNA output of each promoter in 1000 cells. We measured the correlation coefficient between the two promoters at multiple time points in the simulation (Table S5). As stated in the text, the low correlation coefficients in Table S5 imply that noise in *comK* is intrinsic, and fluctuations in extrinsic factors such as the number of Spo0A molecules per cell does not have a large effect on whether the cell transitions to competence.

1. Gillespie D (1977) Exact stochastic simulation of coupled chemical reactions. J Phys Chem 81: 2340-2361.
2. Hambraeus G, von Wachenfeldt C, Hederstedt L (2003) Genome-wide survey of mRNA half-lives in Bacillus subtilis identifies extremely stable mRNAs. Mol Genet Genomics 269: 706-714.
